# THC exposure during adolescence increases impulsivity-like behavior in adulthood in a WIN 55,212-2 self-administration mouse model

**DOI:** 10.3389/fpsyt.2023.1148993

**Published:** 2023-05-25

**Authors:** María del Mar Cajiao-Manrique, Verònica Casadó-Anguera, Alejandra García-Blanco, Rafael Maldonado, Elena Martín-García

**Affiliations:** ^1^Laboratory of Neuropharmacology-Neurophar, Department of Medicine and Life Sciences, Universitat Pompeu Fabra (UPF), Barcelona, Spain; ^2^Hospital del Mar Medical Research Institute (IMIM), Barcelona, Spain; ^3^Departament de Psicobiologia i Metodologia de les Ciències de la Salut, Universitat Autònoma de Barcelona (UAB), Barcelona, Spain

**Keywords:** cannabis addiction, adolescence, WIN 55,212-2 self-administration mouse model, THC, impulsivity, *drd2*, *adora2a*

## Abstract

**Background:**

Cannabis addiction is a chronically relapsing disorder lacking effective treatment. Regular cannabis consumption typically begins during adolescence, and this early cannabinoid exposure may increase the risk for drug addiction in adulthood.

**Objective:**

This study investigates the development of cannabis addiction-like behavior in adult mice after adolescent exposure to the main psychoactive component of cannabis, Δ^9^-tetrahydrocannabinol (THC).

**Methods:**

Adolescent male mice were exposed to 5 mg/kg of THC from postnatal days 37 to 57. Operant self-administration sessions of WIN 55,212-2 (12.5 μg/kg/infusion) were conducted for 10 days. Mice were tested for three addiction-like criteria (persistence of response, motivation, and compulsivity), two parameters related to craving (resistance to extinction and drug-seeking behavior), and two phenotypic vulnerability traits related to substance use disorders (impulsivity and reward sensitivity). Additionally, qPCR assays were performed to detect differentially expressed genes in medial prefrontal cortex (mPFC), nucleus accumbens (NAc), dorsal striatum, and hippocampus (HPC) of “addicted” and “non-addicted” mice.

**Results:**

Adolescent THC exposure did not modify WIN 55,212-2 reinforcement nor the development of cannabis addiction-like behavior. Inversely, THC pre-exposed mice displayed impulsive-like behavior in adulthood, which was more pronounced in mice that developed the addiction-like criteria. Moreover, downregulated *drd2* and *adora2a* gene expression in NAc and HPC was revealed in THC pre-exposed mice, as well as a downregulation of *drd2* expression in mPFC of vehicle pre-treated mice that developed addiction-like behaviors.

**Discussion:**

These findings suggest that adolescent THC exposure may promote impulsivity-like behavior in adulthood, associated with downregulated *drd2* and *adora2a* expression in NAc and HPC.

## 1. Introduction

*Cannabis sativa* derivatives have become the most widely used illicit drugs worldwide, and regular cannabis use mainly begins during adolescence. Indeed, it is estimated that 15.5% of EU inhabitants aged 15–34 used cannabis in 2021, while 19.1% of those aged 15–24 had consumed cannabis in the last year and 10.4% in the last month (1). With a recent increase in the prevalence of cannabis use disorder (CUD) and a reduction in the perceived risk of cannabis use (2), it is necessary to further understand the neurobiological consequences associated with this cannabis consumption. However, the consequences of early-life cannabis use are still poorly understood. Recent evidence indicates that early exposure to Δ^9^-tetrahydrocannabinol (THC), the primary psychotropic constituent of cannabis, produces changes in the structure and function of brain circuits implicated in decision-making and cognitive processes (3). However, whether such changes might be long-lasting and persistently disrupt healthy behaviors remains unknown.

The adolescent period is a critical phase of brain development, neuronal maturation, and restructuring (4–6). Imaging studies have reported that higher-order structures, such as the prefrontal cortex, are still immature at that time point (7, 8) and they cannot fully inhibit behavior favoring impulsivity and risk-taking (9). Additionally, the striatal and limbic circuits are hyperactive during adolescence, leading to greater emotional reactivity and reward-seeking behaviors (7). These studies propose that early-life cannabis exposure may impair neurodevelopment and induce changes that affect the adult brain (8). Thus, a loss of gray matter was revealed in the prefrontal areas of adolescent cannabis users (10). Moreover, THC mediates its pharmacological effect through the activation of the endocannabinoid (eCB) system, a key modulatory system crucial in synapse pruning, formation, and maturation (10, 11). Therefore, dysregulation of the eCB system by the action of THC or other exogenous cannabinoids during adolescence could disrupt normal brain development and function. These alterations have been demonstrated to be involved in the earlier onset of psychiatric disorders (6, 10, 12). In addition, a gateway hypothesis has been postulated, proposing a causal chain sequence in which cannabis consumption would be used prior to other illicit drugs and cannabis use would increase the likelihood of using other illicit drugs (9, 13, 14). However, studies on the long-term neurobiological consequences of cannabis consumption in juveniles are scarce and often contradictory.

Cannabis addiction can be conceptualized as a three-stage recurring cycle of “binge/intoxication,” “withdrawal/negative affect,” and “preoccupation/anticipation,” each sustained by neurobiological alterations mainly in the basal ganglia, extended amygdala, and prefrontal cortex, respectively (15). According to the *Diagnostic and Statistical Manual of Mental Disorders* (DSM-5), these stages are characterized by (1) dysregulation of rewarding or pleasurable effects, (2) emergence of a negative emotional state when access to the reward is prevented, and (3) loss of control in limiting drug intake despite repeated unsuccessful efforts to resist, respectively (8, 15). These stages are repeated and worsen over time, perpetuating the addiction cycle until they produce a maladaptive habit formation that is governed by compulsive behavior. Moreover, craving was included in the DSM-5 addiction diagnostic criteria as it is directly related to the vulnerability to relapse after abstinence (16, 17). In our mouse model of cannabis addiction, we study these three stages through the three hallmarks of addiction based on the DSM-5 addiction criteria: (1) persistence of drug-seeking, (2) motivation for the drug, and (3) compulsive-like behavior. Moreover, we also evaluate two parameters related to craving, resistance to extinction and drug-seeking behavior after abstinence, and two phenotypic traits considered to be factors of vulnerability to substance misuse, impulsivity and sensitivity to reward. According to its multifactorial origin, only a subset of individuals repeatedly exposed to the drug will develop addiction (9, 18, 19).

In this study, we aim to investigate the development of cannabis addiction-like behavior and related behavioral alterations in adult mice after adolescent exposure to THC. For this purpose, adolescent mice received daily administration of THC or vehicle from postnatal days (PND) 37 to 57, considered to be the adolescent period in mice, equivalent to 12–19 years old in humans (20). Then, mice were trained to acquire an operant intravenous (iv) self-administration conditioning sustained by WIN 55,212-2, a potent synthetic cannabinoid agonist (21). The addiction-like criteria were evaluated, as well as parameters related to craving and phenotypic traits, as previously described. We also evaluated molecular markers of cannabis addiction-like behavior in brain areas of the mesocorticolimbic circuit.

## 2. Materials and methods

### 2.1. Animals

Eight weeks old male C57BL/6J mice (*n* = 40) (Charles River, France) were housed individually in controlled laboratory conditions (21 ± 1°C, 55 ± 10%) with food and water available *ad libitum*. The male sex was chosen considering previous literature that has validated the operant WIN 55,212-2 self-administration model only in males (22). Mice were tested during the first hours of the dark phase of a reversed light/dark cycle (lights off at 8:00 a.m. and on at 20:00 p.m.). Body weight and food intake were monitored throughout the experiment. All animal procedures were approved by the local ethical committee (Comitè Ètic d’Experimentació Animal-Parc de Recerca Biomèdica de Barcelona, CEEA-PRBB, agreement N°9687) and conducted strictly in accordance with the guidelines of the European Communities Council Directive (2010/63/EU) regulating animal experimentation, in the animal facility at Universitat Pompeu Fabra-Barcelona Biomedical Research Park (UPF-PRBB; Barcelona, Spain). All the experiments were performed under blind and randomized conditions.

### 2.2. Drugs

Dronabinol, Δ-9-tetrahydrocannabinol (THC), generously gifted by Rhodes Pharmaceuticals, Coventry, RI, USA, stored at 100 mg/ml in sesame oil, was dissolved in a vehicle containing 5% ethanol 100%, 5% cremophor-LE (C5135, Sigma-Aldrich, USA) and 90% physiological saline solution, and administered by intraperitoneal (ip) injection at a dose of 5 mg/kg of body weight. For the self-administration, WIN 55, 212-2 [(R)-(+)-WIN 55,212-2 mesylate salt, Sigma-Aldrich, USA] was dissolved in one drop of Tween 80 (TWEEN 80, Sigma-Aldrich, USA) and then diluted in heparinized (1%) sterile saline solution and made available at 0.1 mg/kg for ip injection 24 h before the first operant session and 12.5 μg/kg/infusion for the self-administered iv infusions. The preparation was covered from the light and stored at room temperature. After each self-administration session, 0.05 ml of sodic heparin (Hospira 5%, Hospira, Pfizer) was applied through the iv catheter to avoid coagulation and obstruction of the latter. Thiopental sodium (5 mg/ml, Braun Medical S.A.) was dissolved in distilled water and delivered in a volume of 0.05 ml through the iv catheter.

### 2.3. Operant self-administration apparatus

Experiments were performed in mouse operant chambers (model ENV-307A-CT, Med Associates Inc., Georgia, VT, USA) equipped with two nose-poke holes, one randomly selected as the active hole and the other as the inactive hole. A house light was located on the ceiling of the chamber, and two stimuli lights (cues) were located one inside the active hole and the other above it. Nose-poking in the active hole resulted in the delivery of one WIN 55,212-2 infusion (under the associated schedule) paired with the activation of the stimulus light located above the active hole, while nose-poking in the inactive hole had no consequences. The chambers were made of aluminum and acrylic and were housed inside sound- and light-attenuated boxes equipped with fans that provided ventilation and white noise. The chamber’s floor was a grid made of metal bars that could conduct electrical current when performing the shock test. WIN 55,212-2 was delivered in a volume of 23.5 μl over 2 s via a syringe mounted on a microinfusion pump (PHM-100A, Med-Associates, Georgia, VT, USA) and connected with flexible polymer tubing (0.96 mm outer diameter, Portex Fine Bore Polythene Tubing, Portex Limited, Kent, United Kingdom) to a single channel liquid swivel (375/25, Instech Laboratories, Plymouth Meeting, PA, USA) and to the mouse iv catheter.

### 2.4. Experimental design

Adolescent mice received a daily dose of 5 mg/kg of THC or vehicle from PND 37 to PND 57. Afterward, mice were implanted with an intrajugular catheter in order to perform iv drug administrations. Subsequently, mice were trained to acquire an operant drug self-administration conditioning maintained by iv infusions of WIN 55,212-2 under a fixed-ratio (FR) 1 schedule of reinforcement during five sessions, followed by five sessions of a FR2 schedule of reinforcement. After the training, three addiction-like criteria resembling DSM-5 criteria for addiction, persistence to response, motivation, and compulsive-like behavior, two parameters related to craving, resistance to extinction and drug-seeking behavior, and two phenotypic traits considered factors of vulnerability to substance misuse, impulsivity and sensitivity to reward, were evaluated in each mouse (Supplementary Figure 3).

### 2.5. Adolescent THC treatment

We evaluated the long-term effect of THC exposure during adolescence on WIN 55,212-2 self-administration, reinstatement, addiction-like criteria, and phenotypic traits present in adulthood. Mice were divided into two groups and administered daily with THC (*n* = 20) or its vehicle (*n* = 20) at a dose of 5 mg/kg of body weight from PND 37 to PND 57, corresponding to the adolescent period in mice based on previous literature (23). This dose of THC was chosen according to previous studies using similar doses to translate in rodents the doses used for smoked cannabis in humans (24–27). Body weight and food intake were strictly monitored for the entire period of treatment. Subsequently, animals underwent surgical procedures, and once adulthood was reached (PND 68), self-administration experiments were started.

### 2.6. WIN 55,212-2 self-administration

#### 2.6.1. Jugular vein catheterization

Mice were anesthetized by ip injection (0.2 ml/10 g of body weight) of ketamine hydrochloride (75 mg/kg of body weight, Ketamidor, Richter Pharma AG, Austria) and medetomidine hydrochloride (1 mg/kg of body weight, Domtor, Esteve, Spain) dissolved in 0.9% physiological saline and then implanted with indwelling iv catheters in the right jugular vein, as previously described (22). Briefly, a 6 cm long silicone tubing (0.3 mm inner diameter, 0.6 mm outer diameter; Silastic, Dow Corning, Houdeng-Goegnies, Belgium) was adapted to a 22-gauge steel cannula (Semat, Herts, United Kingdom) angled at a right angle and then embedded in a dental cement disk (Dentalon Plus, Heraeus Kulzer, Germany) with an underlying nylon mesh. The catheter tubing was inserted 1.1 cm into the right jugular vein and attached with a suture, and the remaining tubing was placed subcutaneous (sc) to the cannula, exiting at the midscapular region. All incisions were sutured and coated with a local analgesic (Blastoestimulina, Almirall, Spain). After, a post-surgery procedure consisting of an ip injection of antibiotic (1 mg/kg of body weight, Gentamicine, Genta-Gobens, Laboratorios Normon, Spain), a sc injection of analgesic (mixture of glucose serum (GlucosaVet, B. Braun Vet Care, Spain) and meloxicam (2 mg/kg of body weight, Metacam, Boehringer Ingelheim, Rhein) and a sc injection of anesthesia reverser, atipamezole hydrochloride (2.5 mg/kg of body weight, Revertor, Virbac, Spain) was applied, all dissolved in sterile 0.9% physiological saline. Mice were allowed to recover for 3 days with follow-up analgesics prior to the initiation of the self-administration sessions. The patency of iv catheters was evaluated by a thiopental sodium test at the end of the self-administration experimental sequence. If prominent signs of anesthesia were not observed immediately after injection, the mouse was removed from the experiment.

#### 2.6.2. WIN 55,212-2 self-administration training

The operant model was applied similarly to previous drug self-administration paradigms (22, 28) with the inclusion of the addiction-like criteria, parameters related to craving and phenotypic traits. To avoid the aversive effects to the drug’s first administration, mice received an ip injection of WIN 55,212-2 24 h before the first self-administration session. Subsequently, mice were trained to acquire operant self-administration maintained by iv infusions of WIN 55,212-2. The schedule was a fixed ratio (FR) 1 schedule of reinforcement (one active nose-poking resulted in one drug delivery) during five consecutive sessions, followed by a progression to FR2 (two active nose-pokings resulted in one drug delivery) for another five sessions. All sessions were performed at the same time every day. Each daily self-administration session initiated with a priming injection of the drug, followed by two 55 min active periods separated by a 15 min drug-free period. The beginning of each operant session was signaled by turning on the house-light only during the first 3 s. During the active periods, each drug infusion was paired contingently with the cue light located above the active nose-poke under the specific FR schedule. These cue lights, together with the noise of the infusion pump, acted as environmental cues signaling the drug infusion. A 10 s time-out period was fixed after each drug delivery, during which the cue light was off and no infusion was provided after responding to the active nose-poke. Responses to the active and inactive holes and all responses executed during the time-out period were recorded. During the drug-free period, neither reinforcer nor contingent cue light was delivered, and this period was signaled by the illumination of the whole self-administration chamber. The session was concluded after 50 reinforcers were delivered or after 125 min, whichever occurred first. If a mouse reached 50 reinforcers, the limit was expanded to 100 reinforcers in the following session. The acquisition of the self-administration behavior was achieved when all of the following conditions were met: (1) mice maintained 80% stability in three consecutive sessions, that is, the variance across these 3 days was 20% or less, (2) at least 75% responding in the active hole, and (3) a minimum of five reinforcers per session. After each session, mice were brought back to their home cages.

#### 2.6.3. Three addiction-like criteria

The development of addictive-like behaviors was evaluated at the end of the training sessions based on three addiction-like criteria that summarize the addiction hallmarks according to the DSM-5 (29, 30). The addiction-like score developed was then attributed based on the results of these three criteria, determined by the respective behavioral test:

##### 2.6.3.1. Persistence of response

Non-reinforced active responses during the 15 min drug-free period were measured as persistence of drug-seeking behavior. Mice were scored on the three consecutive days before the progressive ratio (PR).

##### 2.6.3.2. Motivation

The PR schedule of reinforcement evaluated the motivation for WIN 55,212-2. The responses required to receive one single drug infusion escalated following this series: 1, 5, 12, 21, 33, 51, 75, 90, 120, 155, 180, 225, 260, 300, 350, 410, 465, 540, 630, 730, 850, 1,000, 1,200, 1,500, 1,800, 2,100, 2,400, 2,700, 3,000, 3,400, 3,800, 4,200, 4,600, 5,000, and 5,500. The breaking point, the maximal number of responses the animal performs to obtain one infusion, defined as the motivation value, corresponds to the last ratio completed. The duration of the PR session was maximum 4 h-long or until mice stopped responding to any nose-poke within 1 h.

##### 2.6.3.3. Compulsivity

Resistance to punishment, defined as compulsive-like behavior, corresponded to the maintenance of active responding behavior despite its association with a negative consequence. It was measured by the total number of shocks obtained in a 50 min shock test, during which each drug delivered was associated with a foot-shock-induced punishment. This shock session was performed after a stabilizing FR2 self-administration session following the PR test. Mice were placed in a different operant box than the one usually used for operant training. Then, mice underwent a FR2 self-administration schedule of reinforcement for 50 min composed of two schedule changes: after nose-poking once in the active hole, mice received an electric foot-shock (0.18 mA, 2 s), while if they performed a second active nose-poke, the electric foot-shock was paired with the drug delivery and the associated cue light. After the time-out period that follows drug delivery, the latter schedule was reinitiated. In parallel, if the second response was not completed within a min after completing the first response, the sequence was also reinitiated.

#### 2.6.4. Establishment of mice subpopulations

After performing the three behavioral tests, mice were categorized into “addicted” and “non-addicted” animals based on the number of achieved positive criteria. A mouse was considered positive for an addiction-like criterion when the score of the behavioral test was equal to or beyond the 75th percentile of the normal distribution of the vehicle group. Mice that achieved 2 or 3 criteria were considered “addicted” and categorized as vulnerable, whereas those reaching 0 or 1 criteria were considered “non-addicted” and categorized as resilient.

#### 2.6.5. Extinction and parameters related to craving

Only mice with patent catheters that achieved all acquisition criteria continued to the extinction phase. After thiopental testing, mice were allowed to rest for 1 day, during which they underwent a 2-h locomotion test in individual locomotor activity boxes (10.8 × 20.3 × 18.6 cm, Imetronic, Pessac, France) equipped with infrared sensors to detect locomotor activity and an infrared plane to detect rearings.

During the extinction period, neither WIN 55,212-2 infusions nor the associated environmental cues were delivered after nose-poking in the active hole. Mice were exposed to 2-h daily sessions for 12 consecutive days in the same operant chamber as the self-administration sessions. During this period, the extinction criterion was achieved when active responses were <35% of the mean responses obtained during the last 3 days of WIN 55,212-2 self-administration across three consecutive extinction sessions. Only mice that met the extinction criterion were evaluated for reinstatement. Two parameters related to craving were evaluated before and after this extinction period:

##### 2.6.5.1. Resistance to extinction

Number of active responses in 2 h during the first extinction session. Animals with significant sensitivity to drug withdrawal would increase their resistance to extinction by enhancing the number of active nose-pokes to seek the drug when access is prevented for 2 h the first time after the training sessions (31–33).

##### 2.6.5.2. Drug-seeking behavior measured by cue-induced reinstatement of WIN 55,212-2-seeking behavior

The day after reaching the extinction criterion, we performed a single cue-induced reinstatement test in the same operant chamber, in order to test reinstatement of drug-seeking behavior upon exposure to the environmental stimuli. The cue test was conducted under the same conditions used in the acquisition phase, except that active responding was not reinforced by the drug. Mice were exposed to a 90-min FR2 session, where the first 60 min were similar to an extinction session, but in the last 30 min, nose-poking in the active hole led to the presentation of all associated environmental cues (cue light, pump noise, and priming injection light), but not the delivery of WIN 55,212-2.

#### 2.6.6. Behavioral tests to evaluate addiction-like phenotypic traits

Two additional phenotypic traits were also evaluated as factors of vulnerability to addiction-like behavior:

##### 2.6.6.1. Impulsivity

Non-reinforced active responses during the time-out periods (10 s) after each drug delivery were used as measures of impulsivity-like behavior, which indicated the inability to end a response once it is initiated. The three consecutive days before the PR test were considered for this criterion. Mice were then categorized as high-impulsive (HI) (score above the median) or low-impulsive (LI) (score below the median), as previously described (34, 35). The different subpopulations were split according to the median since impulsivity data was not normally distributed.

##### 2.6.6.2. Sensitivity to reward

The number of reinforcers obtained in 2-h sessions during the last three consecutive FR2 operant conditionings maintained by WIN 55,212-2. Animals with higher levels of sensitivity to reward will obtain a higher number of reinforcers.

### 2.7. Gene expression analysis

#### 2.7.1. Tissue preparation

Tissue collection was performed immediately after the cue-induced reinstatement test. Mice were euthanized by cervical dislocation. Immediately afterward, brains were extracted from the skull and processed rapidly on ice. The medial prefrontal cortex (mPFC), nucleus accumbens (NAc), dorsal striatum (DS), and hippocampus (HPC) were isolated according to the following coordinates from the Paxinos and Franklin atlas (36): (mPFC) AP +1.98 mm; (NAc) AP +1.94 mm; (DS) AP +0.62 mm; (HPC) AP −2.92 mm. Samples were placed in individual tubes, frozen on dry ice and stored at −80°C until RNA isolation. The remaining brain parts from the same animals were also frozen on dry ice and stored at −80°C.

#### 2.7.2. RNA extraction

Total RNAs from the mPFC, NAc, DS, and HPC were extracted using TRIzol™ (Thermo Fisher Scientific, USA) for subsequent RT-PCR analysis. Briefly, tissues were homogenized in TRIzol reagent and, after adding chloroform, the aqueous phase was collected and incubated in isopropanol to isolate the RNA. Samples were kept in dry ice meanwhile to ensure their integrity. Tubes were then centrifuged and pellets were resuspended in 75% ethanol, before another centrifugation. Supernatants were carefully discarded to air dry the pellet for at least 2 h. Finally, pellets were resuspended in RNase-free water, incubated for 15 min at 55–60°C and stored at −80°C. RNA concentrations were quantified by Nanodrop (NanoDrop One, Thermo Fisher Scientific, USA).

#### 2.7.3. Quantitative polymerase chain reaction (qPCR)

The quantitative conversion of the extracted RNA into single-stranded complementary DNA (cDNA) was performed using random primers included in the High Capacity cDNA Reverse Transcription (RT) kit (Applied Biosystems, 4390778, Thermo Fisher Scientific, USA). Real-time PCR analysis was carried out with the following primers (Sigma-Aldrich, USA): *drd1*: forward “AGATTGACCAGGAAGAGGCC,” reverse “GCAATCCAAGCCATACCAGG”; *drd2*: forward “CCATCTC TTGCCCACTGCTCTTTGG,” reverse “GGTGACGATGAAGGG CACGTAGAAC”; *adora2a*: forward “CGTCACCAACTT CTTCGTGG,” reverse “GCTGAAGATGGAACTCTGCG”; *cnr1*: forward “CCTGGGAAGTGTCATCTTTGT,” reverse “GGTAACCCCACCCAGTTTGA.” These primers were used in combination with the PowerSYBR Green PCR MasterMix kit (Applied Biosystems, Thermo Fisher Scientific, USA). Assays were analyzed with the QuantStudio™ 12K Flex real-time PCR system (Applied Biosystems, Thermo Fisher Scientific, USA). Relative expression of mRNAs was determined after normalization with a housekeeping gene using the ΔΔCt method. All validated differentially expressed genes were normalized using β-actine. The gene expression of β-actine was measured as housekeeping gene in these samples using quantitative polymerase chain reaction (qPCR) to verify that the expression of these genes was not affected by the operant model used.

### 2.8. Statistics

#### 2.8.1. Statistical analysis of behavioral data

The number of mice (*n*) in each experimental condition is indicated in figure legends. All statistical comparisons were performed with SPSS (IBM, version 25). Comparisons between two groups were performed by Student’s *t*-test or U Mann–Whitney test depending on the distribution defined by the Shapiro–Wilk test. ANOVA with repeated measures (or Friedman test when non-parametric) was employed when required to test the evolution over time, with subsequent *post hoc* analysis (Fisher LSD). Two-way ANOVA with subsequent *post hoc* analysis (Tukey) was used for multiple group comparisons. The Pearson correlation coefficient was performed to analyze the relationship between values in each addiction-like criterion and the final criteria achieved. The Chi-square analysis was used to compare the percentage of “addicted” with the “non-addicted” mice. Results were expressed as individual values with the median and the interquartile range or with the mean ± SEM, specified in the figure legend. A *p*-value < 0.05 was used to determine statistical significance.

The sample size was calculated based on the power analysis. The significance criterion (alpha) was set at 0.050 and the statistical test used was the two-sample *t*-test. With the sample size of 14–16 mice per group, our studies achieved a power superior to 80%. Supplementary Tables 1–7 give details of the statistical results for the data presented in the figures.

#### 2.8.2. Principal component analysis

The principal component analysis (PCA) was performed to evaluate the multidimensional behavioral data by reducing it to fewer dimensions to observe trends, jumps, clusters, and outliers. PCA and varimax rotation were conducted using the three addiction-like criteria, the two parameters related to craving and the two phenotypic traits considered as vulnerability factors of addiction-like behavior, and dimensionality was reduced to the minimum number of components that best explain and maximize the variance present in the data set. An eigenvalue greater than 1 was set as the selecting criterion.

## 3. Results

### 3.1. Effects of THC pre-treatment during adolescence on the operant model of cannabinoid addiction-like behavior using WIN 55,212-2 self-administration training in adulthood

**FIGURE 1:**
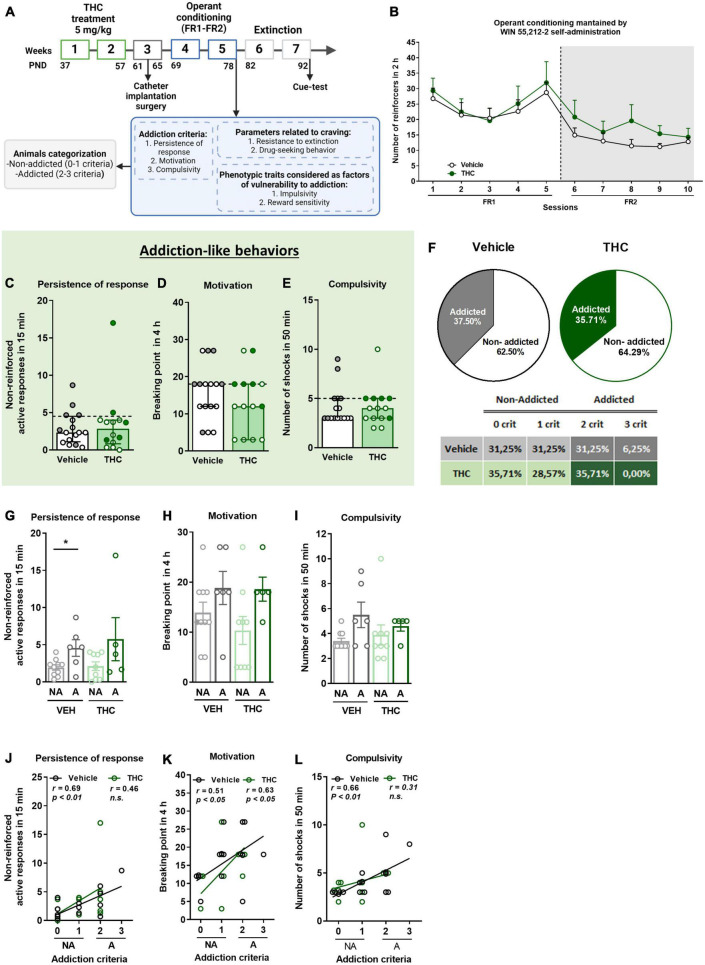
THC administration during adolescence led to the development of an addictive-like phenotype after WIN 55,212-2 operant self-administration in adult mice. **(A)** Timeline of the experimental sequence of the WIN 55,212-2 self-administration mouse model. **(B)** Number of infusions obtained by vehicle and THC pre-treated groups during 2 h of operant self-administration maintained by intravenous infusions of WIN 55,212-2 under both FR1 and FR2 schedules of reinforcement (mean ± SEM, repeated measures ANOVA). **(C–E)** Mice presented similar responses in the three addiction-like criteria tests (individual data with median and interquartile range). **(C)** Persistence of response: number of active nose-poke responses during the 15 min drug-free period (U Mann–Whitney). **(D)** Motivation: breaking point determined during a 4 h progressive schedule of reinforcement represents the maximal number of responses that an animal is able to emit to obtain one drug infusion (Student’s *t*-test). **(E)** Compulsivity: number of shocks received following the schedule described in section “Materials and methods”, reflecting the compulsivity level of each group (U Mann–Whitney). The dashed horizontal line indicates the 75th percentile of the distribution of the group, used as the threshold to consider a mouse positive for one criterion. “Addicted” mice are represented in gray-filled circles for the vehicle group and green-filled circles for the THC group. **(F)** Percentage of mice categorized as “addicted” (Chi-square). **(G–I)** Behavioral tests of the three addiction-like criteria when separated between “addicted” and “non-addicted” (individual data with median and interquartile range, Student’s *t*-test for persistence and motivation, U Mann–Whitney for compulsivity, *p < 0.05). Pearson correlations between individual values of addiction-like criteria, and **(J)** non-reinforced active responses in 15 min, **(K)** breaking point in 4 h, and **(L)** number of shocks in 50 min (nVehicle = 16, nTHC = 14; statistical details are included in Supplementary Table 1).

We explored the effects of THC exposure in adolescent mice on specific behavioral signatures of cannabis addiction-like behavior in adulthood. Mice were trained to acquire an operant self-administration conditioning maintained by iv infusions of WIN 55,212-2 under an FR1 schedule of reinforcement during 5 sessions followed by 5 sessions under FR2, for a total of 10 consecutive sessions (Figure 1A). Under the FR1 schedule, no significant differences were found between THC and vehicle pre-treated mice, suggesting similar reinforcing effects of WIN 55,212-2 (Repeated measures ANOVA, *F*_(1_,_28)_ = 0.00, n.s., Figure 1B). When the requirement was increased to FR2, THC pre-treated mice showed a tendency to obtain a higher number of reinforcers (mean ± SEM of the last three operant sessions = 15.7 ± 3.18) compared to vehicle pre-treated mice (mean ± SEM of the last three operant sessions = 11.81 ± 1.31), which was maintained until the end of the sessions (Repeated measures ANOVA, *F*_(1_,_28)_ = 1.00, n.s.). The acquisition of operant learning was equivalent between groups (percentage of mice having acquired the behavior: 58.82% of vehicle and 50% of THC, Chi-square, C-S = 0.45, n.s.).

### 3.2. Mouse model of addiction-like behavior using WIN 55,212-2 self-administration and THC pre-treatment during adolescence

After the operant training, the addiction-like behaviors were evaluated, as explained above. No significant differences were found between THC and vehicle pre-treated mice in any of the addiction-like criteria, namely, persistence of response, motivation, and compulsivity (Figures 1C–E). As expected, extreme subpopulations that present a high persistence of response, motivation, and compulsivity were observed in both groups. Additionally, positive correlations between the number of responses to each addiction-like criterion (non-reinforced active responses, breaking point or number of shocks) and the number of addiction-like criteria achieved were found in the vehicle group, while the THC group presented only a positive correlation for the motivation (Pearson correlations, *p* < 0.05, *p* < 0.01, Figures 1J–L), where “addicted” mice showed the highest values. Mice individually classified into the “addicted” group following the previously described criteria were 37.5% in the vehicle pre-treated group and 35.71% in mice receiving THC during adolescence (Chi-square, C-S = 0.019, n.s., Figure 1F). Based on this classification, addiction-like behaviors were compared, revealing that vehicle pre-treated “addicted” mice had higher persistence of response than “non-addicted” mice (Student’s *t*-test, *t* = −2.72, *p* < 0.05, Figure 1G), whereas no significant differences were found between “addicted” and “non-addicted” mice in terms of motivation and compulsivity regardless of the treatment (Figures 1H, I).

### 3.3. THC pre-exposure during adolescence decreased the resistance to extinction in adulthood

**FIGURE 2:**
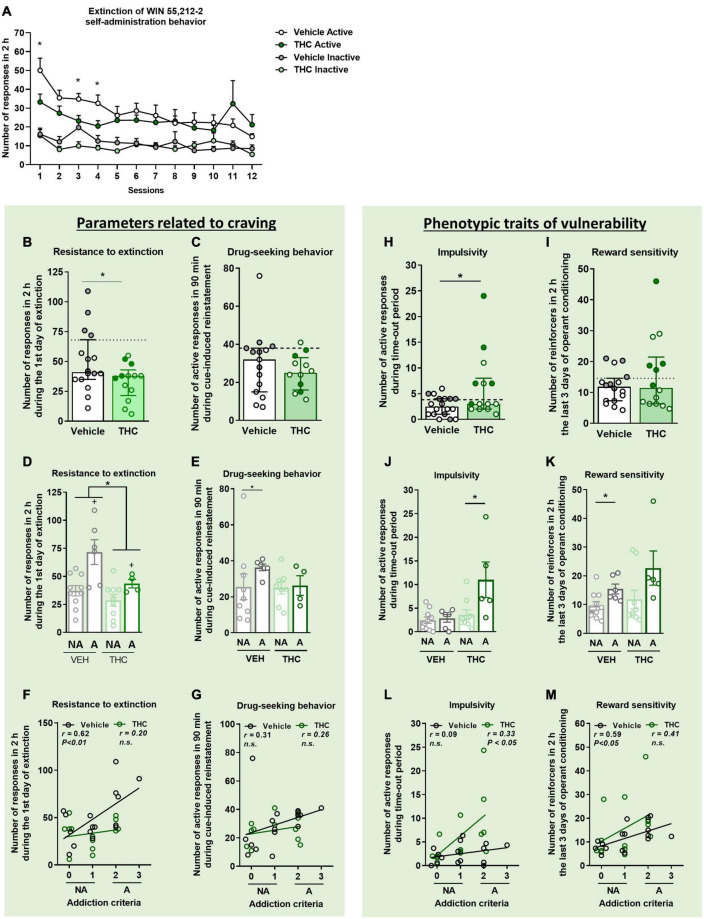
THC administration during adolescence favored the parameters related to craving and the phenotypic vulnerability traits to addiction-like behavior after WIN 55,212-2 operant self-administration in adult mice. **(A)** Extinction pattern of the WIN 55,212-2 self-administration behavior (mean ± SEM, repeated measures ANOVA). **(B,C)** Behavioral tests of the two parameters related to craving (individual data with median and interquartile range). **(B)** Resistance to extinction: number of active nose-poke responses during the first 2-h extinction session is significantly higher in vehicle compared to THC pre-treated mice (Student’s *t*-test, *p < 0.05). **(C)** Drug-seeking behavior with the cue-induced reinstatement after abstinence: number of active responses performed during the 90 min cue-induced drug-seeking test performed after extinction (Student’s *t*-test). The dashed horizontal line indicates the 75th percentile of distribution of the group, used as the threshold to consider a mouse positive for one criterion. “Addicted” mice are represented in gray-filled circles for the vehicle group and green-filled circles for the THC group. **(D,E)** Behavioral tests regarding the parameters related to craving when separating between “addicted” and “non-addicted” mice (individual data with mean ± SEM). **(D)** In resistance to extinction, an effect of THC pre-treatment was detected between vehicle and THC groups as well as an effect of phenotype between “addicted” and “non-addicted” mice (two-way ANOVA, Treatment effec*t* = *p < 0.05, Phenotype effec*t* = ^+^*p* < 0.01). **(E)** Higher drug-seeking behavior was observed in “addicted” vehicle pre-treated animals compared to “non-addicted” (U Mann–Whitney, *p < 0.05). Pearson correlations between individual values of addiction-like criteria, and **(F)** number of responses in 2 h during the first day of extinction and **(G)** number of responses in 90 min during the cue-induced reinstatement test. **(H,I)** Behavioral tests used to evaluate phenotypic traits considered to be factors of vulnerability to addiction-like behavior (individual data with median with interquartile range). **(H)** Impulsivity: number of responses to the active nose-poke during the 10 s time-out period (Student’s *t*-test, *p < 0.05). **(I)** Reward sensitivity: number of reinforcers performed to the active nose-poke during the 2 h of the last three sessions of self-administration (U Mann–Whitney). **(J,K)** Behavioral tests of the phenotypic traits showing higher reward sensitivity in “addicted” mice pre-treated with vehicle compared to “non-addicted”, whereas this difference was observed in the THC group for impulsivity (individual data with mean ± SEM, U Mann–Whitney, *p < 0.05). **(L,M)** Pearson correlations between individual values of addiction-like criteria, and **(L)** number of active responses during time-out period and **(M)** number of reinforcers in 2 h in the last three consecutive days of operant training (nVehicle = 16, nTHC = 14; statistical details are included in Supplementary Table 2).

Mice then underwent 12 sessions of extinction. The extinction trend of both groups was similar, indicating that both displayed the same ability to extinguish the self-administration behavior (Repeated measures ANOVA, *F*_(1_,_26)_ = 1.20, n.s., Figure 2A). A total of 26.7% of vehicle and 8.3% of THC pre-treated mice (Chi-square, C-S = 2.06, n.s.) acquired the extinction criteria, as previously described.

Two parameters closely related to craving and reinstatement of drug-seeking were evaluated. First, the pattern of activity on the first extinction day was evaluated every 10 min as a measurement of resistance to extinction (Supplementary Figure 2). All groups highly responded to the previous active nose-poke at the beginning of the session, meaning that mice were seeking the reinforcer and the cue light associated with it. However, as non-reinforced sessions continued, we see that this behavior diminished, which is characteristic of an extinction behavior. THC pre-treated mice responded less during the first extinction session than vehicle pre-treated mice (Student’s *t*-test, *t* = −2.18, *p* < 0.05, Figure 2B). In a two-way ANOVA analysis, this global treatment effect was also observed between THC and vehicle pre-treated mice. When separating groups into “addicted” and “non-addicted,” an increased resistance to extinction was observed in “addicted” mice compared to “non-addicted” [two-way ANOVA, Treatment effect: *F*(1,25) = 6.872, *p* < 0.05, Phenotype effect: *F*(1,25) = 12.52, *p* < 0.01, Interaction: *F*(1,25) = 1.998, n.s., Figure 2D]. In the vehicle group, active nose-pokes in the first session were very high and decreased across sessions until reducing by 70% in the last session compared to the first one, whereas THC pre-treated mice decreased only by 30% (Figure 2A). In addition, active nose-poking by both groups was significantly higher than inactive nose-poking (Repeated measures ANOVA, *F*_(1_,_48)_ = 15.26, *p* < 0.001, *post-hoc* DMS test: Actives vs. Inactives: *p* < 0.001 in all the comparisons). On the first, third and fourth extinction sessions, vehicle pre-treated mice showed less extinction responding compared to mice pre-treated with THC (Student’s *t*-test, *p* < 0.05).

The reinstatement of drug-seeking behavior was evaluated after mice achieved extinction. No significant differences were found between vehicle and THC pre-treated groups regarding the cue-induced reinstatement of drug-seeking behavior. Only a significant difference was revealed when comparing “addicted” and “non-addicted” mice pre-treated with vehicle. Indeed, “addicted” mice pre-treated with vehicle presented higher responses than vehicle “non-addicted” mice (U Mann–Whitney, *U* = 10.000, *p* < 0.05) (Figures 2C–E).

### 3.4. THC exposure during adolescence increased impulsivity-like behavior in adulthood

Two additional phenotypic traits considered as factors of vulnerability to substance misuse were evaluated. THC pre-treated mice displayed higher impulsivity-like behavior compared to vehicle pre-treated mice (Student’s *t*-test, *t* = −2.17, *p* < 0.05, Figure 2H), an effect that was more pronounced in “addicted” mice pre-treated with THC (U Mann–Whitney, *U* = 6.500, *p* < 0.05, Figure 2J). Contrary, no significant differences were found between vehicle and THC pre-treated groups for the reward sensitivity during the last 3 days of operant training (Figure 2I), although “addicted” mice pre-treated with vehicle had more reward sensitivity compared to “non-addicted” (U Mann–Whitney, *U* = 10.000, *p* < 0.05, Figure 2K). These results were emphasized by the positive correlations found between the number of responses to each addiction-like criterion and the number of addiction-like criteria achieved (Pearson correlations, Figures 2F, G, L, M).

### 3.5. High- and low-impulsive subgroups reveal behavioral differences in adult mice after WIN 55,212-2 self-administration

**FIGURE 3:**
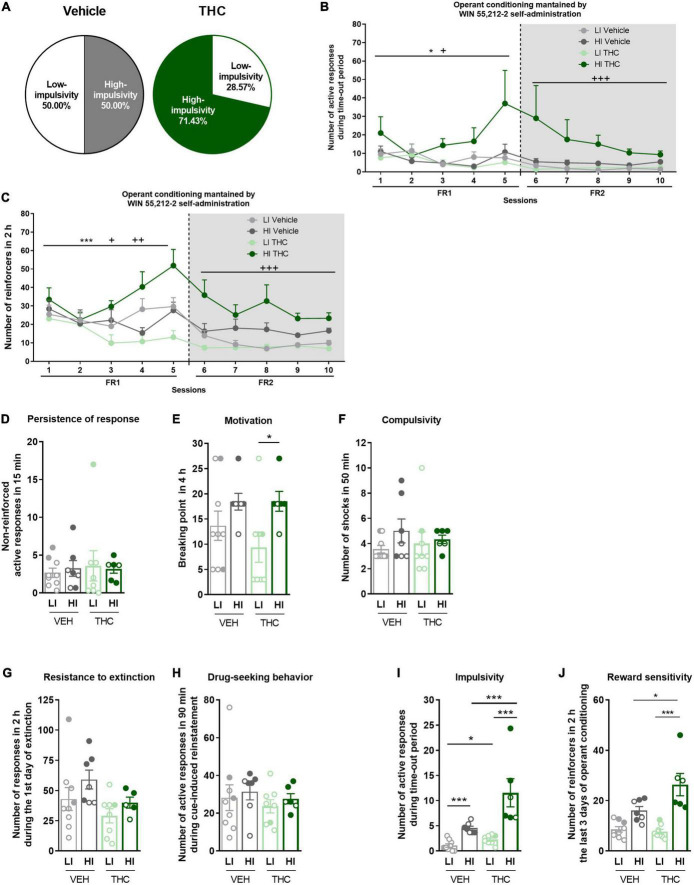
Characterization of low-impulsive (LI) and high-impulsive (HI) subgroups. **(A)** Percentage of mice categorized as LI and HI (Chi-square). **(B)** HI mice pre-treated with THC showed a higher number of responses during the time-out period under both FR1 and FR2 schedules compared to HI mice pre-treated with vehicle, as well as to LI mice (repeated measures ANOVA, *p < 0.05 session effect in FR1, ^+^*p* < 0.05 treatment effect in FR1, ^+++^*p* < 0.001 treatment effect in FR2). **(C)** HI mice pre-treated with THC showed higher acquisition of the operant conditioning under FR2 schedule compared to HI mice pre-treated with vehicle, as well as to LI mice (Repeated measures ANOVA, ***p < 0.001 session effect in FR1, ^++^*p* < 0.01 treatment × session effect in FR1, ^+^*p* < 0.05 treatment effect in FR1; ^+++^*p* < 0.001 treatment effect in FR2). **(D–F)** Behavioral tests of the three addiction-like criteria when separated between LI and HI (mean ± SEM, U Mann–Whitney, *p < 0.05). **(G–J)** Behavioral tests of the **(G,H)** two parameters related to craving (Student *t*-test or *U* Mann–Whitney) and **(I,J)** the two phenotypic vulnerability traits when separated between LI and HI (mean ± SEM, impulsivity: Student *t*-test or *U* Mann–Whitney, reward sensitivity: two-way ANOVA with Tukey’s multiple comparisons test, *p < 0.05, ***p < 0.001) (nVehicle = 16, nTHC = 14; statistical details are included in Supplementary Table 3).

To further investigate the enhanced impulsive behavior promoted by THC pre-exposure, mice were categorized into high-impulsive and low-impulsive subgroups defined according to the median (see section “Materials and methods”). We find 50.0% of high-impulsive mice in the vehicle group and 71.43% in the THC group (Chi-square, C-S = 2.571, n.s., Figure 3A). High-impulsive mice pre-treated with THC showed a higher number of active responses during the time-out period compared to high-impulsive mice pre-treated with vehicle, as well as to low-impulsive mice under both the FR1 (Repeated measures ANOVA, *F*_(1_,_26)_ = 3.46, *p* < 0.05, *post-hoc* DMS test: HI THC vs. HI vehicle: *p* < 0.05, HI THC vs. LI vehicle: *p* < 0.05, Figure 3B) and FR2 schedules (Repeated measures ANOVA, *F*_(1_,_26)_ = 7.86, *p* < 0.001, *post-hoc* DMS test: HI THC vs. HI vehicle: *p* < 0.01, HI THC vs. LI vehicle: *p* < 0.001). Moreover, high-impulsive mice pre-treated with THC obtained a higher number of reinforcers during the operant conditioning when tested under the FR1 schedule compared to THC pre-treated low-impulsive mice (Repeated measures ANOVA, *F*_(1_,_26)_ = 3.44, *p* < 0.05, *post-hoc* DMS test: HI THC vs. LI THC: *p* < 0.01, Figure 3C), as well as to high-impulsive mice pre-treated with vehicle during sessions 4 (U Mann–Whitney, *U* = 1.000, *p* < 0.01) and 5 (U Mann–Whitney, *U* = 7.000, *p* < 0.05). Also, high-impulsive mice pre-treated with THC presented higher responses in the FR2 schedule compared to high-impulsive mice pre-treated with vehicle and to both low-impulsive mice (Repeated measures ANOVA, *F*_(1_,_26)_ = 12.73, *p* < 0.001, *post-hoc* DMS test: HI THC vs. HI vehicle: *p* < 0.01, HI THC vs. LI vehicle: *p* < 0.001, and HI THC vs. LI THC: *p* < 0.001). Furthermore, high-impulsive mice presented higher motivation compared to low-impulsive mice only in the THC pre-treated group, whereas no significant results were revealed for other addiction-like criteria (U Mann–Whitney, *U* = 7.000, *p* < 0.05, Figures 3D–F).

No significant differences were revealed in the parameters related to craving (Figures 3G, H). Both low-impulsive (U Mann–Whitney, *U* = 15.000, *p* < 0.05) and high-impulsive mice (U Mann–Whitney, *U* = 0.000, *p* < 0.001) receiving THC during adolescence show more extreme values of impulsivity compared to those pre-treated with vehicle (Figure 3I). As expected, high-impulsive mice have higher impulsivity than low-impulsive mice regardless of the treatment (U Mann–Whitney, *U* = 0.000, *p* < 0.001 for vehicle, and U Mann–Whitney, *U* = 0.000, *p* < 0.001 for THC). In terms of reward sensitivity, a phenotypic difference was found between low-impulsive and high-impulsive mice, as well as an effect of the treatment and of the interaction between both [two-way ANOVA, Phenotype effect: *F*(1,26) = 39.38, *p* < 0.001, Treatment effect: *F*(1,26) = 5.194, *p* < 0.05, Interaction: *F*(1,26) = 7.314, *p* < 0.05, Figure 3J]. High-impulsive animals pre-treated with THC showed higher reward sensitivity compared to low-impulsive mice pre-treated with THC (Tukey’s multiple comparisons, *p* < 0.001), as well as to high-impulsive mice pre-treated with vehicle (Tukey’s multiple comparisons, *p* < 0.05).

### 3.6. THC pre-treatment did not produce major long-term behavioral or somatic alterations

Δ^9^-Tetrahydrocannabinol exposure has been reported to produce a series of behavioral and somatic alterations (37). Several behavioral and somatic responses (body weight, food intake, and locomotor activity) were monitored throughout the experiment in order to verify that THC pre-treatment during adolescence at the dose of 5 mg/kg did not produce any long-term effect in adulthood that could bias our self-administration protocol. No significant differences were observed between THC and vehicle pre-treated mice in body weight and food intake during the whole operant sequence, even though the weight gain of mice pre-treated with THC was apparently lower than that of vehicle pre-treated mice during the adolescent treatment (Supplementary Figures 1A–C). This is in accordance with previous studies employing similar protocols that have shown that THC treatment may reduce weight gain in adolescent mice compared to vehicle pre-treated animals (27, 38). Moreover, no significant differences were observed between groups in the number of beam breaks per 10 min during 120 min (Supplementary Figure 1D), which supports the absence of major behavioral alteration promoted by THC pre-exposure.

### 3.7. Principal component analysis of THC exposure during adolescence in self-administration of WIN 55,212-2 in adulthood

**FIGURE 4:**
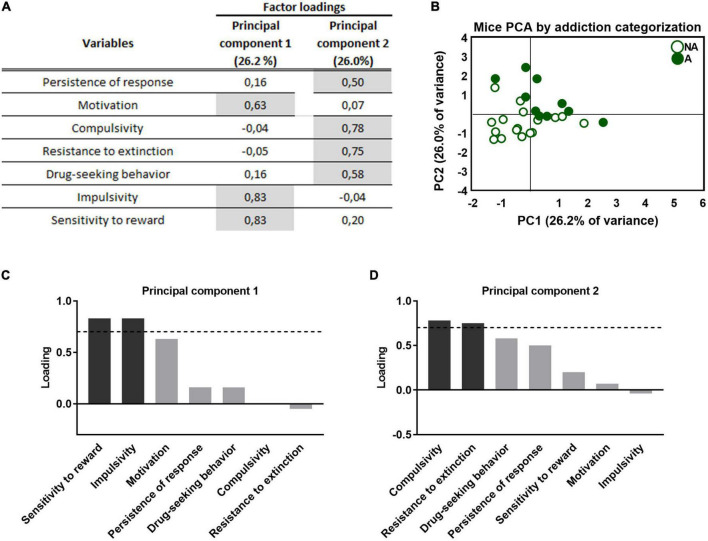
Principal component analysis of the effect of adolescent THC exposure in WIN 55,212-2 operant self-administration in adulthood. **(A)** Factor loadings of principal component 1 (PC1) and principal component 2 (PC2) for all variables studied. **(B)** Individual mice clustered according to addiction or non-addiction in the space yielded by the 2 PCA components, which accounted for the maximum data variance, with factor loadings of 26.2% for PC1 and 26.0% for PC2. **(C,D)** Order of factor loading of the different variables in PC1 and PC2. The dashed horizontal line marks loading greater than 0.7, mainly contributing to the component. In regards to the addiction-like criteria, a dissociation between motivation, mainly contributing to PC1, and compulsivity, mainly contributing to PC2, can be observed. Moreover, impulsivity and reward sensitivity weighted more in the PC1, while resistance to extinction and drug-seeking weighted more in the PC2.

Principal component analysis was used to characterize whether the behavioral outcomes previously described could be reduced to fewer dimensions. All addiction-like criteria, parameters related to craving, and phenotypic traits were taken into account. This analysis separated two clear clusters representing the separation between the “addicted” and “non-addicted” groups and both populations present clear behavioral differences that allow for a distinction (Figure 4B). Component 1 accounts for 26.2% of the variance (Figures 4A, B) and has strong loadings (>0.7) from three behavioral variables: motivation, impulsivity, and reward sensitivity. The second component is orthogonal to component 1, accounts for 26.0% of the variance, and comprises four variables: the criteria of persistence of response, compulsivity, resistance to extinction, and drug-seeking behavior. Impulsivity participates more in the first component, while compulsivity is more critical in the second component (Figures 4C, D), resembling the sequential feature of the transition from impulsivity to compulsivity that has been described in addiction (39). Also, motivation and compulsivity belong to different components, as the neural substrate of each addiction criterion is different (15).

### 3.8. Correlation heatmap between the addiction-like criteria, parameters related to craving and vulnerability phenotypic traits

**FIGURE 5:**
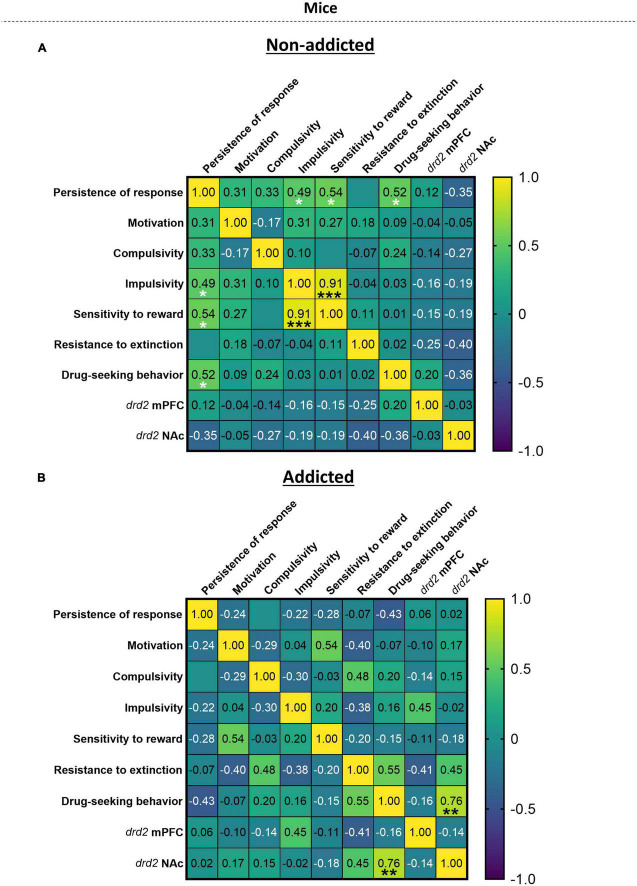
Correlation heatmap of the variables of cannabis addiction-like criteria, parameters related to craving and vulnerability phenotypic traits. **(A,B)** Pearson correlations between the three addiction-like criteria, the two parameters related to craving and the two phenotypic traits in both **(A)** “non-addicted” and **(B)** “addicted” groups (Pearson correlation, *p < 0.05, **p < 0.01, ***p < 0.001).

When representing the addiction-like criteria, parameters related to craving and phenotypic traits in a heat map (Figure 5), we revealed significant correlations in “non-addicted” animals between response persistence and impulsivity (*r* = 0.49, *p* < 0.05), persistence of response and sensitivity to reward (*r* = 0.54, *p* < 0.05), persistence of response and drug-seeking behavior (*r* = 0.52, *p* < 0.05), and sensitivity to reward and impulsivity (*r* = 0.91, *p* < 0.05). In “addicted” animals, a significant correlation was revealed between drug-seeking behavior and *drd2* NAc (*r* = 0.76, *p* < 0.01).

### 3.9. THC exposure during adolescence produced a downregulation of the *drd2* and *adora2a* gene expression in the NAc and HPC

**FIGURE 6:**
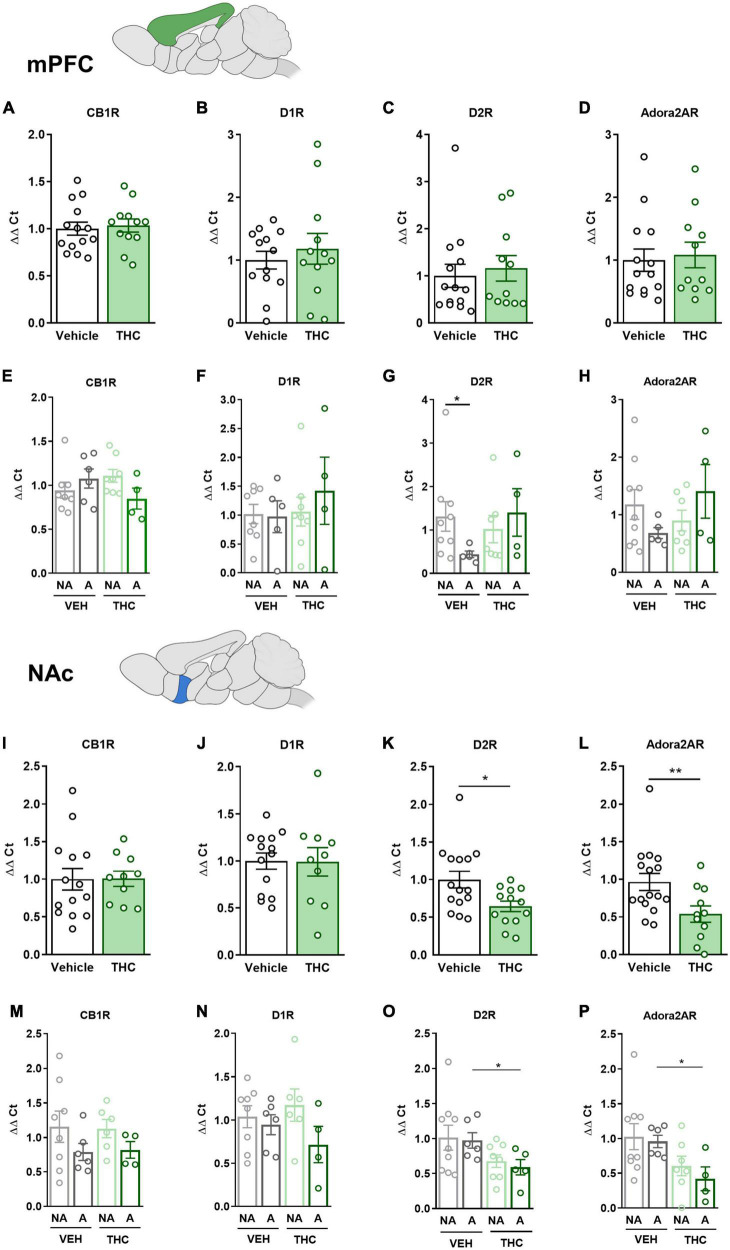
*drd1*, *drd2*, *adora2a*, and *cnr1* gene expression in the medial prefrontal cortex (mPFC) and nucleus accumbens (NAc) after adolescent THC exposure in WIN 55,212-2 operant self-administering adult mice. Gene expression measured by the ΔΔCt after RT-qPCR of *cnr1*, *drd1*, *drd2*, and *adora2a* in the PFC: **(A–D)** between vehicle and THC pre-treated mice respectively (mean ± SEM, Student’s *t*-test for CB1R and D1R, U Mann–Whitney for D2R and Adora2AR), **(E–H)** between “addicted” and “non-addicted” mice respectively (mean ± SEM, Student’s *t*-test or U Mann–Whitney, *p < 0.05). Gene expression measured by the ΔΔCt after qPCR of *cnr1*, *drd1*, *drd2*, and *adora2a* in the NAc: **(I–L)** between vehicle and THC pre-treated mice respectively (mean ± SEM, Student’s *t*-test, *p < 0.05, **p < 0.01), **(M–P)** between “addicted” and “non-addicted” mice respectively (mean ± SEM, Student’s *t*-test or U Mann–Whitney, *p < 0.05) (nVehicle = 16, nTHC = 14; statistical details are included in Supplementary Table 4).

**FIGURE 7:**
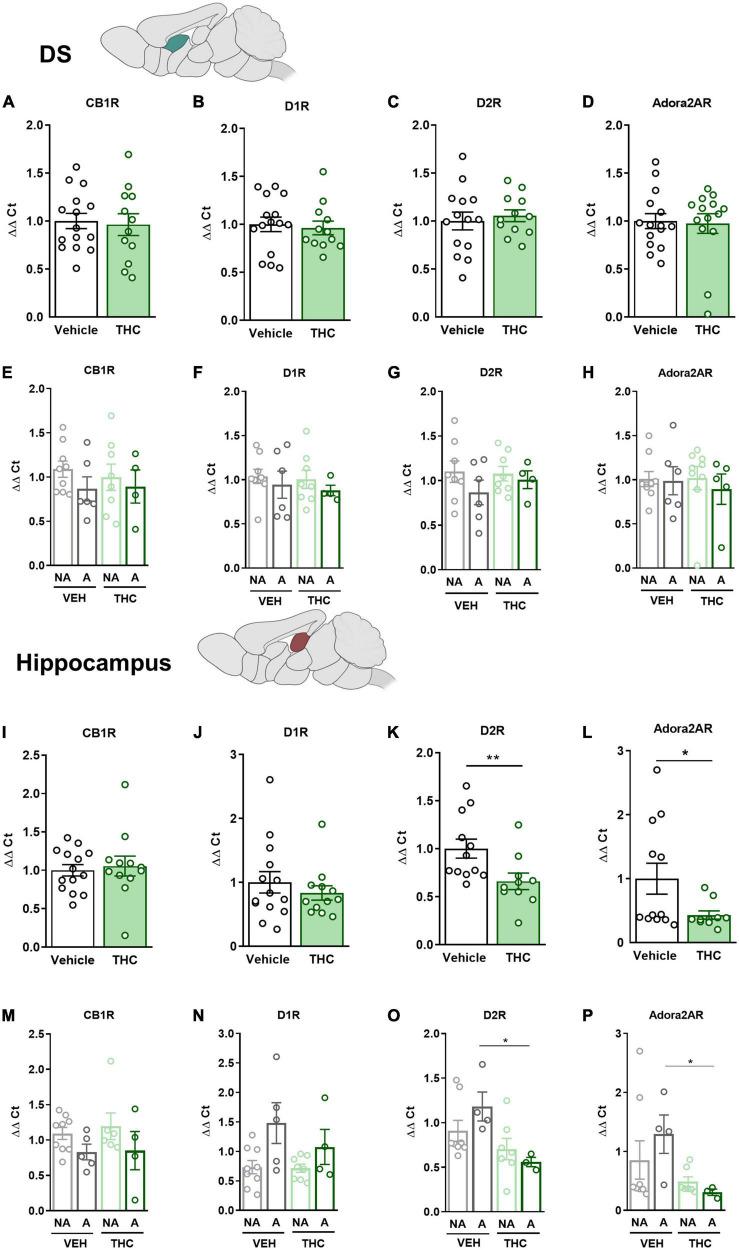
*drd1*, *drd2*, *adora2a*, and *cnr1* gene expression in the dorsal striatum (DS) and hippocampus (HPC) after adolescent THC exposure in WIN 55,212-2 operant self-administering adult mice. Gene expression measured by the ΔΔCt after RT-qPCR of *cnr1*, *drd1*, *drd2*, and *adora2a* in the DS: **(A–D)** between vehicle and THC pre-treated mice respectively (mean ± SEM, Student’s *t*-test for CB1R, D2R, and Adora2AR, U Mann–Whitney for D1R), **(E–H)** between “addicted” and “non-addicted” mice respectively (mean ± SEM, Student’s *t*-test). Gene expression measured by the ΔΔCt after qPCR of *cnr1*, *drd1*, *drd2*, and *adora2a* in the NAc: **(I–L)** between vehicle and THC pre-treated mice respectively (mean ± SEM, Student’s *t*-test for CB1R, D1R, and D2R, U Mann–Whitney for Adora2AR, *p < 0.05, **p < 0.01), **(M–P)** between “addicted” and “non-addicted” mice respectively (mean ± SEM, Student’s *t*-test or U Mann–Whitney, *p < 0.05) (nVehicle = 16, nTHC = 14; statistical details are included in Supplementary Table 5).

The mPFC, NAc, DS, and HPC were extracted at the end of the operant evaluation to study potential neurobiological alterations promoted by THC pre-exposure during adolescence. In a previous study, the expression of *drd1*, *drd2*, and *adora2a* genes was found to be upregulated in food- and cocaine-addicted mice after operant self-administration paradigms (40). Moreover, the *cnr1* gene has been revealed to be involved in addictive disorders (41). Consequently, RT-qPCR assays were performed to compare the differential gene expression of these candidate genes in areas of the mesocorticolimbic circuit. No significant differences between THC and vehicle pre-treated mice were revealed in the mPFC (Figures 6A–D). However, a downregulation in the expression of the *drd2* gene was observed in “addicted” mice pre-treated with vehicle compared to “non-addicted” in this brain area (U Mann–Whitney, *U* = 6.000, *p* < 0.05, Figure 6G). No significant differences were found for *cb1* (Figure 6E), *drd1* (Figure 6F) or *adora2a* (Figure 6H). In the NAc, *drd2* and *adora2a* expression was significantly downregulated in THC pre-treated mice compared to vehicle (Student’s *t*-test, *t* = 2.60, *p* < 0.05 and *t* = 2.83, *p* < 0.01, respectively, Figures 6K, L), and these differences were specifically found in “addicted” mice (Student’s *t*-test, *t* = 2.44, *p* < 0.05 and *t* = 3.10, *p* < 0.05, respectively, Figures 6O, P). Similarly, *drd2* and *adora2a* expression was downregulated in THC pre-treated mice in the HPC (Student’s *t*-test, *t* = 2.54, *p* < 0.01 and U Mann–Whitney, *U* = 31.000, *p* < 0.05, respectively, Figures 7K, L), and these differences were specifically found in “addicted” mice (Student’s *t*-test, *t* = 3.18, *p* < 0.05 and *t* = 3.06, *p* < 0.05, respectively, Figures 7O, P). No significant differences were found for *cb1* or *drd1* in the NAc (Figures 6I, J, M, N) or the HPC (Figures 7I, J, M, N). No significant differences were found in the DS (Figures 7A–H).

## 4. Discussion

In this study, we assessed the effects of THC exposure during adolescence on the vulnerability to develop cannabis addiction-like behavior and related behavioral alterations in adulthood by using a WIN 55,212-2 self-administration mouse model. We found that chronic exposure to THC from PND 37 to 57 increased impulsivity in adult mice, which was more pronounced in the subgroup of mice that developed the addiction-like criteria. Furthermore, THC treatment during adolescence increased the percentage of high-impulsive mice and favored the reinforcement of WIN 55,212-2 in this group. Moreover, high-impulsive mice pre-treated with THC presented an increased reward sensitivity compared to low-impulsive mice pre-treated with THC, as well as to high-impulsive mice pre-treated with vehicle. Inversely, pre-exposure to THC during adolescence decreased the resistance to extinguishing the operant behavior.

Impulsivity is a complex construct defined as a “predisposition toward rapid, unplanned reactions to internal and external stimuli without regard for the negative consequences of these reactions to themselves or others” (42) that is composed of motor and choice impulsivity (43–47). Motor impulsivity is described as the inability to inhibit behavior by changing the course of action or to stop a response once it is initiated (48). We have defined motor impulsivity as the non-reinforced active nose-pokes during the time-out periods that reflect the motor disinhibition, as previously described (47). Compulsivity has its roots in the signs associated with obsessive-compulsive disorder and might comprise repetitive behaviors in the face of adverse consequences after the loss of control (49). As such, we implemented a punishment-based behavioral test where perseverance in responding despite punishment defined compulsive behavior. Finally, reward sensitivity represents the tendency of individuals to approach a positive reinforcer (50). In this article, it is operationally defined as the mean of drug infusions obtained during the last three sessions of operant training.

Δ^9^-Tetrahydrocannabinol pre-treatment during adolescence increased impulsivity in adulthood. Although impulsive behavior may be a pre-existing trait that promotes the usage of drugs, acute and chronic cannabis consumption may result in behavioral changes, including increases in impulsivity, which may facilitate further drug use or the transition to use other drugs of abuse according to the gateway theory (51). In humans, marijuana use has been associated with increased impulsivity when given in an acute (52) or chronic manner (53). Concomitantly, individuals with CUD have been shown to present increased impulsivity (54). Interestingly, impulsivity is suggested to predispose compulsivity, which leads to drug abuse and addiction (55, 56). Indeed, it has been shown that high impulsivity predicts the transition from controlled to compulsive cocaine-taking in rats, as high impulsive rats displayed greater resistance to punishment of the cocaine-taking response compared to low impulsive rats, while a correlation analysis revealed, at the population level, that impulsivity predicts compulsivity (56). Compulsivity shares similarities with impulsivity in inhibitory control dysfunction, which relates to alterations in the mPFC networks to subcortical regions. However, impulsivity and compulsivity may be on a continuum, with compulsivity being repetitive and perseverative (57). In our study, THC pre-treatment during adolescence did not increase the percentage of mice reaching cannabinoid addiction-like criteria but increased the levels of impulsivity-like behavior. Furthermore, within this group, “addicted” mice showed the highest values of motor impulsivity compared to the rest of the groups. This result highlights that the interaction between THC adolescent exposure and impulsivity in addiction development should be explored further.

Our results are in accordance with observations suggesting an overrepresentation of highly impulsive individuals with substance abuse disorders and a high comorbidity between impulsive behavior and substance misuse (56). Indeed, the development of drug addiction would represent a progression from initial impulsivity mediated by the NAc to compulsive, habitual responding, a hallmark feature of addiction (49, 56). Accordingly, in our study, PCA analysis separated the constructs of impulsivity from compulsivity and reward sensitivity from resistance to extinction, distinguishing the two extremes in the development continuum of the disorder between the early stage of onset and the late stage of establishment (15). Consequently, exposure to THC during adolescence may increase the vulnerability to developing cannabis addiction by enhancing impulsivity.

High-impulsive mice pre-treated with THC obtained more reinforcers during the operant conditioning sessions than vehicle-treated mice, suggesting increased reinforcing effects of WIN 55,212-2. In the same line, the acquisition of WIN 55,212-2 self-administration was also reported to be enhanced in rats after adolescent THC exposure, suggesting an increased sensitivity to the reinforcing effects of cannabinoid agonists in adulthood (26). However, the relationships between this change and impulsive behavior were not evaluated in this previous study. Moreover, in our study, THC pre-treatment increased the reward sensitivity to WIN 55,212-2 in high-impulsive mice. It has been documented that a history of cannabinoid exposure in adolescent animals may enhance the sensitivity to other drugs of abuse (58), as the positive hedonic experience generated by these early cannabinoid exposures may alter mesocorticolimbic dopaminergic pathways, increasing the later probability of responding to other drugs of abuse (15). Thus, understanding adolescent cannabis use mechanisms is crucial for drug use prevention, considering that several authors have suggested a controversial gateway theory proposing that cannabis users gradually transition into taking more addictive drugs (59–62). Yet, further research is required, as other competing explanations of the association between cannabis use and the use of other illicit drugs have been debated over the last decades (63–65).

THC treatment during adolescence decreased the resistance to the extinction of WIN 55,212-2 self-administration behavior. Resistance to extinction can be observed as an “extinction burst” behavior typically seen in rodents during the first day of extinction (31, 66–68), which is reported to reflect a “craving-like” state (17) directly linked with the vulnerability to relapse after abstinence (16, 17). The eCB system plays a central role in the regulation of learning and memory (69) and THC treatment during adolescence might alter memory processes, leading to this lack of extinction burst that reflects the strength of the previously acquired operant behavior. A body of literature has demonstrated the deleterious impact of THC abuse during adolescence on memory function in adulthood in mice and rats (70–72). Cognitive deficits have also been revealed in human cannabis users after chronic cannabis consumption, associated with sustained CB1R activation, particularly in working memory and cognitive flexibility (73). It is possible that these cognitive deficits may have accounted for the differences in resistance to extinction and impulsivity found in THC pre-treated mice.

Treatment with THC during adolescence produced a downregulation in the expression of the *drd2* gene in the NAc. This is in agreement with previous studies in chronic cannabis users showing a blunted brain reactivity to dopamine-releasing stimulant drugs in the striatum (74, 75), where dopamine responses measured with PET and [^11^C]raclopride in the NAc were inversely correlated with addiction severity and craving (74). This pattern is also shown in the chronic use of other drugs of abuse, like in cocaine-addicted human patients, where a downregulation of the dopamine D2 receptor (D2R) was reported in the NAc after repeated exposure to the drug (76), which has not been revealed to date in cannabis users (62). Reduced D2R expression in the striatum has been linked with enhanced impulsivity (7). Furthermore, low D2R availability in the striatum predicts subsequent levels of cocaine self-administration in rhesus monkeys (77) and human cocaine abusers (55). We suggest that adolescent THC exposure in our study, promoted impulsivity-like behavior in adulthood through the downregulation of *drd2* expression in the NAc. The dopamine system exerts different roles in reinforcement, motivation, and self-regulation, leading to up and downregulated expression of the dopamine receptors as the addiction develops (76). Further research should assess whether the impulsivity trait predates the onset of cannabis use or if the use of cannabis during adolescence produces alterations in the brain.

Downregulation of the *adora2a* gene is also observed in the NAc of mice exposed to THC during adolescence. Adenosine A2A receptors (A2AR) participate in the control of the rewarding and motivational properties of drugs (78). In agreement with our results, the specific involvement of the A2AR in the addictive-related properties of THC has been demonstrated (79). Also, methamphetamine self-administration for 14 days in rats decreased A2AR in the accumbens shell (80), whereas stimulation of A2AR was reported to decrease drug self-administration, primarily through the formation of a heteromer with the D2R (81).

The *drd2* gene is also downregulated in the mPFC of “addicted” mice pre-treated with vehicle compared to “non-addicted.” In agreement, a decrease in *drd2* expression was reported in the mPFC of addicted mice chronically treated with cocaine (82). This downregulation of the *drd2* gene in the mPFC further supports the impulsive phenotype revealed in “addicted” mice.

Pre-treatment with THC downregulated the *drd2* gene in the HPC. Accordingly, cocaine-induced downregulation of the D2R in the HPC promotes spine plasticity that favors conditioned place preference behaviors (83). The downregulation observed in our study after THC during adolescence could alter short- and long-term memory processes necessary for the development of cannabis addiction-like behavior in adulthood. The *adora2a* gene was also downregulated in the HPC, in accordance with literature stating that deficits of A2AR function promote impulsive behavior related to HPC modifications (84). Finally, no changes in *cnr1* gene expression were found in any of the four brain areas evaluated, suggesting that any possible change in CB1R expression promoted by THC exposure during adolescence might be transient.

In this study, the male sex was chosen considering the previous literature on drug addiction models (22, 27). Considering all these studies previously performed in male rodents, further research should now focus on the potential sex differences in the propensity of adolescent THC exposure to increase the sensitivity to other drugs of abuse.

## 5. Conclusion

In summary, this study revealed that THC exposure during adolescence promoted impulsivity in adulthood, increased WIN 55,212-2 reinforcement and favored the sensitivity to reward in high-impulsive mice, and decreased the resistance to extinction. The behavioral changes promoted by adolescent THC exposure were associated with downregulation of *drd2* and *adora2a* expression in NAc and HPC, which may be closely related to the changes revealed in impulsive-like behavior. These behavioral and neurochemical changes reveal long-lasting consequences promoted by THC exposure during adolescence and further emphasize the potential negative consequences related to early consumption of cannabis.

## Data availability statement

The original contributions presented in this study are included in the article/Supplementary material, further inquiries can be directed to the corresponding authors.

## Ethics statement

This animal study was reviewed and approved by the Comitè Ètic d’Experimentació Animal-Parc de Recerca Biomèdica de Barcelona, CEEA-PRBB, agreement N°9687.

## Author contributions

EM-G and RM conceived and designed the animal model and the behavioral approaches associated, and supervised and revised the manuscript. MC-M, VC-A, and AG-B conducted the behavioral experiments, and molecular experiments and analysis, performed the statistical and data analyses, generated the associated graphs, and interpreted the results under the supervision of EM-G and RM. MC-M, VC-A, and EM-G performed the catheterization surgeries. MC-M wrote the manuscript and prepared the figures under the supervision of EM-G and RM. All authors contributed to the article and approved the submitted version.

## References

[1] European Monitoring Centre for Drugs and Drug Addiction. *European Drug Report; Trends and Developments.* Portugal: European Monitoring Centre for Drugs and Drug Addiction (2022). 10.2810/75644

[2] Palacios-Ceña D Jiménez-Trujillo I Hernández-Barrera V Florencio LL Carrasco-Garrido P. Time trends in the co-use of cannabis and the misuse of tranquilizers, sedatives and sleeping pills among young adults in spain between 2009 and 2015. *Int J Environ Res Public Health.* (2019) 16:3423. 10.3390/ijerph16183423 31540173 PMC6765996

[3] Ferland J-MN Ellis RJ Betts G Silveira MM Firmino JB Wistanley C Long-term outcomes of adolescent THC exposure on translational cognitive measures in adulthood in an animal model and computational assessment of human data. *JAMA Psychiatry.* (2022) 80:66–76. 10.1001/jamapsychiatry.2022.3915 36416863 PMC9685552

[4] Casey BJ Tottenham N Liston C Durston S. Imaging the developing brain: what have we learned about cognitive development? *Trends Cogn Sci.* (2005) 9:104–10. 10.1016/j.tics.2005.01.011 15737818

[5] Marsh R Gerber AJ Peterson BS. Neuroimaging studies of normal brain development and their relevance for understanding childhood neuropsychiatric disorders. *J Am Acad Child Adolesc Psychiatry.* (2008) 47:1233–51. 10.1097/CHI.0b013e318185e703 18833009 PMC2759682

[6] Simpson AK Magid V. Cannabis use disorder in adolescence. *Child Adolesc Psychiatr Clin N Am.* (2016) 25:431–43. 10.1016/j.chc.2016.03.003 27338965

[7] Volkow ND Boyle M. Neuroscience of addiction: relevance to prevention and treatment. *Am J Psychiatry.* (2018) 175:729–40. 10.1176/appi.ajp.2018.17101174 29690790

[8] Volkow ND Michaelides M Baler R. The neuroscience of drug reward and addiction. *Physiol Rev.* (2019) 99:2115–40. 10.1152/physrev.00014.2018 31507244 PMC6890985

[9] Compton WM Wargo EM Volkow ND. Neuropsychiatric model of addiction simplified. *Psychiatr Clin North Am.* (2022) 45:321–34. 10.1016/j.psc.2022.05.001 36055726 PMC9450117

[10] Dhein S. Different effects of cannabis abuse on adolescent and adult brain. *Pharmacology.* (2020) 105:609–17. 10.1159/000509377 32629444

[11] Maldonado R Berrendero F Ozaita A Robledo P. Neurochemical basis of cannabis addiction. *Neuroscience.* (2011) 181:1–17. 10.1016/j.neuroscience.2011.02.035 21334423

[12] Hurd YL Manzoni OJ Pletnikov MV Lee FS Bhattacharyya S Melis M. Cannabis and the developing brain: insights into its long-lasting effects. *J Neurosci.* (2019) 39:8250–8. 10.1523/JNEUROSCI.1165-19.2019 31619494 PMC6794936

[13] Kandel D. Stages in adolescent involvement in drug use. *Science.* (1975) 190:912–4. 10.1126/science.1188374 1188374

[14] Fergusson DM Boden JM Horwood LJ. Cannabis use and other illicit drug use: testing the cannabis gateway hypothesis. *Addiction.* (2006) 101:556–69. 10.1111/j.1360-0443.2005.01322.x 16548935

[15] Koob G Volkow ND. Neurobiology of addiction: a neurocircuitry analysis. *Lancet Psychiatry.* (2016) 3:760–73. 10.1016/S2215-0366(16)00104-8 27475769 PMC6135092

[16] Venniro M Caprioli D Shaham Y. Animal models of drug relapse and craving: from drug-priming induced reinstatement to incubation of craving after voluntary abstinence. *Prog Brain Res.* (2016) 224:25–52. 10.1016/bs.pbr.2015.08.004 26822352

[17] Fredriksson I Venniro M Reiner DJ Chow JJ Bossert JM Shaham Y. Animal models of drug relapse and craving after voluntary abstinence: a review. *Pharmacol Rev.* (2021) 73:1050–83. 10.1124/pharmrev.120.000191 34257149 PMC11060480

[18] Piazza PV Deroche-Gamonet V. A multistep general theory of transition to addiction. *Psychopharmacology.* (2013) 229:387–413. 10.1007/s00213-013-3224-4 23963530 PMC3767888

[19] Hamilton PJ Nestler EJ. Epigenetics and addiction. *Curr Opin Neurobiol.* (2019) 59:128–36. 10.1016/j.conb.2019.05.005 31255844 PMC6889055

[20] Flurkey K Currer JM Harrison DE. Mouse models in aging research. *Mouse Biomed Res.* (2007) III:637–72.

[21] Compton DR Gold LH Ward SJ Balster RL Martin BR. Aminoalkylindole analogs: cannabimimetic activity of a class of compounds structurally distinct from delta 9-tetrahydrocannabinol. *J Pharmacol Exp Ther.* (1992) 263:1118–26. 1335057

[22] Mendizábal V Zimmer A Maldonado R. Involvement of kappa/dynorphin system in WIN 55,212-2 self-administration in mice. *Neuropsychopharmacology.* (2006) 31:1957–66. 10.1038/sj.npp.1300957 16292318

[23] Schneider M. Adolescence as a vulnerable period to alter rodent behavior. *Cell Tissue Res*. (2013) 354:99–106. 10.1007/s00441-013-1581-2 23430475

[24] Wiley JL O’Connell MM Tokarz ME Wright MJ Jr. Pharmacological effects of acute and repeated administration of Δ9-tetrahydrocannabinol in adolescent and adult rats. *J Pharmacol Exp Ther.* (2007) 320:1097–105. 10.1124/jpet.106.108126 17172531

[25] Rubino T Vigano D Realini N Guidali C Braida D Capurro V Chronic Δ9-tetrahydrocannabinol during adolescence provokes sex-dependent changes in the emotional profile in adult rats: behavioral and biochemical correlates. *Neuropsychopharmacology.* (2008) 33:2760–71. 10.1038/sj.npp.1301664 18172430

[26] Scherma M Dessì C Muntoni AL Lecca S Satta V Luchicchi A Adolescent Δ 9-Tetrahydrocannabinol exposure Alters WIN55,212-2 self-administration in adult rats. *Neuropsychopharmacology* (2016) 41:1416–26. 10.1038/npp.2015.295 26388146 PMC4793126

[27] Flores A Maldonado R Berrendero F. THC exposure during adolescence does not modify nicotine reinforcing effects and relapse in adult male mice. *Psychopharmacology.* (2020) 237:801–9. 10.1007/s00213-019-05416-8 31858159

[28] Vallée M Vitiello S Bellocchio L Hébert-Chatelain E Monlezun S Martín-García E Pregnenolone can protect the brain from cannabis intoxication. *Science.* (2014) 343:94–8. 10.1126/science.1243985 24385629 PMC4057431

[29] Deroche-Gamonet V Belin D Piazza PV. Evidence for addiction-like behavior in the rat. *Science.* (2004) 30586:1014–7. 10.1126/science.1099020 15310906

[30] Domingo-Rodriguez L Ruiz de Azua I Dominguez E Senabre E Serra I A specific prelimbic-nucleus accumbens pathway controls resilience versus vulnerability to food addiction. *Nat Commun.* (2020) 11:782. 10.1038/s41467-020-14458-y 32034128 PMC7005839

[31] Cooper J Heron T Heward W. *Applied Behavior Analysis.* New York, NY: Macmillan (1987).

[32] Lerman DC Iwata BA. Prevalence of the extinction burst and its attenuation during treatment. *J Appl Behav Anal.* (1995) 28:93–4. 10.1901/jaba.1995.28-93 16795857 PMC1279794

[33] Cooper J Heron T Heward W. *Applied Behavior Analysis.* Upper Saddle River, NJ: Pearson Education (2007).

[34] Piazza PV Deminière JM Le Moal M Simon H. Factors that predict individual vulnerability to amphetamine self-administration. *Science.* (1989) 245:1511–3. 10.1126/science.2781295 2781295

[35] Antoniou K Papathanasiou G Papalexi E Hyphantis T Nomikos GG Spyraki C Individual responses to novelty are associated with differences in behavioral and neurochemical profiles. *Behav Brain Res.* (2008) 187:462–72. 10.1016/j.bbr.2007.10.010 18036673

[36] Franklin K Paxinos G. *The Mouse Brain in Stereotaxic Coordinates.* Cambridge, MA: Academic Press (1997).

[37] Addams IB Martin BR. Cannabis: pharmacology and toxicology in animals and humans. *Addiction.* (1996) 91:1585–614. 8972919

[38] Stopponi S Soverchia L Ubaldi M Cippitelli A Serpelloni G Ciccocioppo R. Chronic THC during adolescence increases the vulnerability to stress-induced relapse to heroin seeking in adult rats. *Eur Neuropsychopharmacol.* (2014) 24:1037–45. 10.1016/j.euroneuro.2013.12.012 24412506

[39] Lüscher C Robbins TW Everitt BJ. The transition to compulsion in addiction. *Nat Rev Neurosci.* (2020) 21:247–63. 10.1038/s41583-020-0289-z 32231315 PMC7610550

[40] Navandar M Martín-García E Maldonado R Lutz B Gerber S Ruiz de Azua I. Transcriptional signatures in prefrontal cortex confer vulnerability versus resilience to food and cocaine addiction-like behavior. *Sci Rep.* (2021) 11:9076. 10.1038/s41598-021-88363-9 33907201 PMC8079697

[41] Benyamina A Kebir O Blecha L Reynaud M Krebs M-O. CNR1 gene polymorphisms in addictive disorders: a systematic review and a meta-analysis. *Addict Biol.* (2011) 16:1–6. 10.1111/j.1369-1600.2009.00198.x 20192949

[42] Moeller FG Barratt ES Dougherty DM Schmitz JM Swann AC. Psychiatric aspects of impulsivity. *Am J Psychiatry.* (2001) 158:1783–93. 10.1176/appi.ajp.158.11.1783 11691682

[43] Koob G Volkow ND. Neurocircuitry of addiction. *Neuropsychopharmacology.* (2010) 35:217–38. 10.1038/npp.2009.110 19710631 PMC2805560

[44] Dalley JW Everitt BJ Robbins TW. Impulsivity, compulsivity and top-down cognitive control. *Neuron* (2011) 69:680–94. 10.1016/j.neuron.2011.01.020 21338879

[45] Belcher AM Volkow ND Moeller FG Ferré S. Personality traits and vulnerability or resilience to substance use disorders. *Trends Cogn Sci.* (2014) 18:211–7. 10.1016/j.tics.2014.01.010 24612993 PMC3972619

[46] Maldonado R Calvé P García-Blanco A Domingo-Rodríguez L Senabre E Martín-García E. Vulnerability to addiction. *Neuropharmacology.* (2021) 186:108466. 10.1016/j.neuropharm.2021.108466 33482225

[47] Domingo-Rodriguez L Cabana-Domínguez J Fernàndez-Castillo N Cormand B Martín-García E Maldonado R. Differential expression of miR-1249-3p and miR-34b-5p between vulnerable and resilient phenotypes of cocaine addiction. *Addict Biol.* (2022) 27:e13201. 10.1111/adb.13201 36001423 PMC9286869

[48] Logan GD Schachar RJ Tannock R. Impulsivity and Inhibitory Control. *Psychol Sci.* (1997) 8:60–4. 10.1111/j.1467-9280.1997.tb00545.x

[49] Everitt BJ Robbins TW. Neural systems of reinforcement for drug addiction: from actions to habits to compulsion. *Nat Neurosci.* (2005) 8:1481–9. 10.1038/nn1579 16251991

[50] Hu W Zhao X Liu Y Ren Y Wei Z Tang Z Reward sensitivity modulates the brain reward pathway in stress resilience via the inherent neuroendocrine system. *Neurobiol Stress.* (2022) 20:100485. 10.1016/j.ynstr.2022.100485 36132434 PMC9483565

[51] De Wit H. Impulsivity as a determinant and consequence of drug use: a review of underlying processes. *Addict Biol.* (2009) 14:22–31. 10.1111/j.1369-1600.2008.00129.x 18855805 PMC3640851

[52] Ansell EB Laws HL Roche MJ Sinha R. Effects of marijuana use on impulsivity and hostility in daily life. *Drug Alcohol Depend.* (2015) 148:136–42. 10.1016/j.drugalcdep.2014.12.029 25595054 PMC4330120

[53] Gruber SA Silveri MM Dahlgren MK Yurgelun-Todd D. Why so impulsive? white matter alterations are associated with impulsivity in chronic marijuana smokers. *Exp Clin Psychopharmacol.* (2011) 19:231–42. 10.1037/a0023034 21480730 PMC3659424

[54] Wagner MF de Oliveria CR Paloski LH. Levels of impulsivity in individuals with cannabis use disorder. *Tends Psychiatry Psychother.* (2022) 44(Suppl. 1):e20210449. 10.47626/2237-6089-2021-0449 35500250 PMC9490940

[55] Dalley JW Fryer TD Brichard L Robinson ESJ Theobald DEH Lääne K Nucleus accumbens D2/3 receptors predict trait impulsivity and cocaine reinforcement. *Science.* (2007) 31516:1267–70. 10.1126/science.1137073 17332411 PMC1892797

[56] Belin D Everitt BJ. Cocaine seeking habits depend upon dopamine-dependent serial connectivity linking the ventral with the dorsal striatum. *Neuron.* (2008) 3:432–41. 10.1016/j.neuron.2007.12.019 18255035

[57] Moore CF Sabino V Koob GF Cottone P. Pathological overeating: emerging evidence for a compulsivity construct. *Neuropsychopharmacology.* (2017) 42:1375–89. 10.1038/npp.2016.269 27922596 PMC5436113

[58] Rubino T Zamberletti E Parolaro D. Adolescent exposure to cannabis as a risk factor for psychiatric disorders. *J Psychopharmacol.* (2012) 26:177–88. 10.1177/0269881111405362 21768160

[59] Yamaguchi K Kandel DB. Patterns of drug use from adolescence to young adulthood: III. predictors of progression. *Am J Public Health.* (1984) 74:673–81. 10.2105/ajph.74.7.673 6742253 PMC1651671

[60] Fergusson DM Horwood LJ. Does cannabis use encourage other forms of illicit drug use? *Addiction.* (2000) 95:505–20. 10.1046/j.1360-0443.2000.9545053.x 10829327

[61] Ellgren M Spano SM Hurd YL. Adolescent cannabis exposure alters opiate intake and opioid limbic neuronal populations in adult rats. *Neuropsychopharmacology.* (2007) 32:607–15. 10.1038/sj.npp.1301127 16823391

[62] Zehra A Burns J Liu CK Manza P Wiers CE Volkow ND Cannabis addiction and the brain: a review. *J Neuroimmune Pharmacol.* (2018) 13:438–52. 10.1007/s11481-018-9782-9 29556883 PMC6223748

[63] Morral AR McCaffrey DF Paddock SM. Reassessing the marijuana gateway effect. *Addiction.* (2002) 97:1493–504. 10.1046/j.1360-0443.2002.00280.x 12472629

[64] Hall WD Lynskey M. Is cannabis a gateway drug? testing hypotheses about the relationship between cannabis use and the use of other illicit drugs. *Drug Alcohol Rev.* (2005) 24:39–48. 10.1080/09595230500126698 16191720

[65] Tarter RE Kirisci L Mezzich A Ridenour T Fishbein D Horner M Does the “gateway” sequence increase prediction of cannabis use disorder development beyond deviant socialization? Implications for prevention practice and policy. *Drug Alcohol Depend.* (2012) 123(Suppl. 1):S72–8. 10.1016/j.drugalcdep.2012.01.015 22365896 PMC3387340

[66] Peltier RL Guerin GF Dorairaj N Goeders NE. Effects of saline substitution on responding and plasma corticosterone in rats trained to self-administer different doses of cocaine. *J Pharmacol Exp Ther.* (2001) 299:114–20. 11561070

[67] Shalev U Grimm JW Shaham Y. Neurobiology of relapse to heroin and cocaine seeking: a review. *Pharmacol Rev.* (2002) 54:1–42. 10.1124/pr.54.1.1 11870259

[68] Soria G Barbano MF Maldonado R Valverde O. A reliable method to study cue-, priming-, and stress-induced reinstatement of cocaine self-administration in mice. *Psychopharmacology* (2008) 199:593–603. 10.1007/s00213-008-1184-x 18488200

[69] Iversen L. Cannabis and the brain. *Brain.* (2003) 126(Pt. 6):1252–70. 10.1093/brain/awg143 12764049

[70] O’Tuathaigh CM Hryniewiecka M Behan A Tighe O Coughlan C Desbonnet L Chronic adolescent exposure to Δ-9-tetrahydrocannabinol in COMT mutant mice: impact on psychosis-related and other phenotypes. *Neuropsychopharmacology.* (2010) 35:2262–73. 10.1038/npp.2010.100 20631688 PMC3055315

[71] Renard J Krebs M-O Jay TM Le Pen G. Long-term cognitive impairments induced by chronic cannabinoid exposure during adolescence in rats: a strain comparisons. *Psychopharmacology.* (2013) 225:781–90. 10.1007/s00213-012-2865-z 22983145

[72] Poulia N Delis F Brakatselos C Polissidis A Koutmani Y Kokras N Detrimental effects of adolescent escalating low-dose Δ9 -tetrahydrocannabinol leads to a specific bio-behavioural profile in adult male rats. *Br J Pharmacol.* (2021) 178:1722–36. 10.1111/bph.15394 33496341

[73] Mizrahi R Watts JJ Tseng KY. Mechanisms contributing to cognitive deficits in cannabis users. *Neuropharmacology.* (2017) 124:84–8. 10.1016/j.neuropharm.2017.04.018 28414051 PMC5540783

[74] Volkow ND Wang G-J Telang F Fowler JS Alexoff D Logan J Decreased dopamine brain reactivity in marijuana abusers is associated with negative emotionality and addiction severity. *Proc Natl Acad Sci USA.* (2014) 111:E3149–56. 10.1073/pnas.1411228111 25024177 PMC4121778

[75] Bloomfield MA Ashok AH Volkow ND Howes OD. The effects of Δ9-tetrahydrocannabinol on the dopamine system. *Nature.* (2016) 53929:369–77. 10.1038/nature20153 27853201 PMC5123717

[76] Volkow ND Wise RA Baler R. The dopamine motive system: implications for drug and food addiction. *Nat Rev Neurosci.* (2017) 18:741–52. 10.1038/nrn.2017.130 29142296

[77] Nader MA Morgan D Gage HD Nader SH Calhoun TL Buchheimer N PET imgaging of dopamine D2 receptors during chronic cocaine self-administration in monkeys. *Nat Neurosci.* (2006) 9:1050–6. 10.1038/nn1737 16829955

[78] Wydra K Gawliński D Gawlińska K Frankowska M Borroto-Escuela DO Fuxe K Adenosine A2AReceptors in substance use disorders: a focus on cocaine. *Cells.* (2020) 9:1372. 10.3390/cells9061372 32492952 PMC7348840

[79] Soria G Castañé A Berrendero F Ledent C Parmentier M Maldonado R Adenosine A2A receptors are involved in physical dependence and place conditioning induced by THC. *Eur J Neurosci.* (2004) 20:2203–13. 10.1111/j.1460-9568.2004.03682.x 15450100

[80] Kavanagh KA Schreiner DC Levis SC O’Neill CE Bachtell RK. Role of adenosine receptor subtypes in methamphetamine reward and reinforcement. *Neuropharmacology.* (2015) 89:265–73. 10.1016/j.neuropharm.2014.09.030 25301277 PMC4250385

[81] Borroto-Escuela DO Wydra K Li X Rodriguez D Carlsson J Jastrzȩbska J Disruption of A2AR-D2R heteroreceptor complexes after A2AR transmembrane 5 peptide administration enhances cocaine self-administration in rats. *Mol Neurobiol.* (2018) 55:7038–48. 10.1007/s12035-018-0887-1 29383683 PMC6061166

[82] Clare K Pan C Kim G Park K Zhao J Volkow ND Cocaine reduces the neuronal population while upregulating dopamine D2-Receptor-Expressing neurons in brain reward regions: sex-effects. *Front Pharmacol.* (2021) 12:624127. 10.3389/fphar.2021.624127 33912043 PMC8072657

[83] Li J Wu Y Xue T He J Zhang L Liu Y Cdc42 signaling regulated by dopamine D2 receptor correlatively links specific brain regions of hippocampus to cocaine addiction. *Biochim Biophys Acta Mol Basis Dis.* (2023) 1869:166569. 10.1016/j.bbadis.2022.166569 36243293

[84] Oliveros A Cho CH Cui A Choi S Lindberg D Hinton D Adenosine A2A receptor and ERK-driven impulsivity potentiates hippocampal neuroblast proliferation. *Transl Psychiatry.* (2017) 7:e1095. 10.1038/tp.2017.64 28418405 PMC5416704

