# Supplementary material for: THC exposure during adolescence increases impulsivity-like behavior in adulthood in a WIN 55,212-2 self-administration mouse model
Source: Front Psychiatry. 2023 May 25;14:1148993. doi: 10.3389/fpsyt.2023.1148993 (PMC10248087; doi:10.3389/fpsyt.2023.1148993)
Supplement: Supplementary file 1 [file Table_1.docx]

Supplementary Material

**THC exposure during adolescence increases impulsivity-like behavior in adulthood in a WIN 55,212-2 self-administration mouse model.**

**María del Mar Cajiao-Manrique, Verònica Casadó-Anguera, Alejandra García-Blanco, Rafael Maldonado^*^, Elena Martín-García^*^.**

***Correspondence**: Corresponding Author: [rafael.maldonado@upf.edu](mailto:rafael.maldonado@upf.edu)

[elena.martin@upf.edu](mailto:elena.martin@upf.edu)

# Supplementary Figures and Tables

## Supplementary Figures

**a**

**d**

**b**

**c**

**SUPPLEMENTARY FIGURE 1. THC pre-treatment did not produce major long-term behavioral or somatic alterations. A-B.** Body weight: Body weight was measured every week during the self-administration protocol. **C.** Food intake: Food intake of both groups was strictly controlled. Mice were fed *ad libitum* with standard pellets, refilled to 95g-100g/animal every two weeks when measurements were made. The average food intake was calculated as the difference between the amount of food refilled in the previous measurement and the amount of food remaining at the moment of the new measurement divided by the number of days between both measurements. **D.** Kinetics of total activity: Total activity was measured by the number of beam breaks represented in 10 min blocks during a 2 h test. (Mean ± S.E.M.; repeated measures ANOVA) (nVehicle=16, nTHC=14; statistical details are included in Supplementary Table S6).

**SUPPLEMENTARY FIGURE 2. Extinction pattern of the first extinction session.** Actives responses are represented into time intervals of 10 minutes separating between non-addicted and addicted animals of the vehicle and THC pre-treated groups **(**Mean ± S.E.M.; Friedman test and U Mann-Whitney, *P<0.05, **P<0.01) (nVehicle=16, nTHC=14; statistical details are included in Supplementary Table S7).


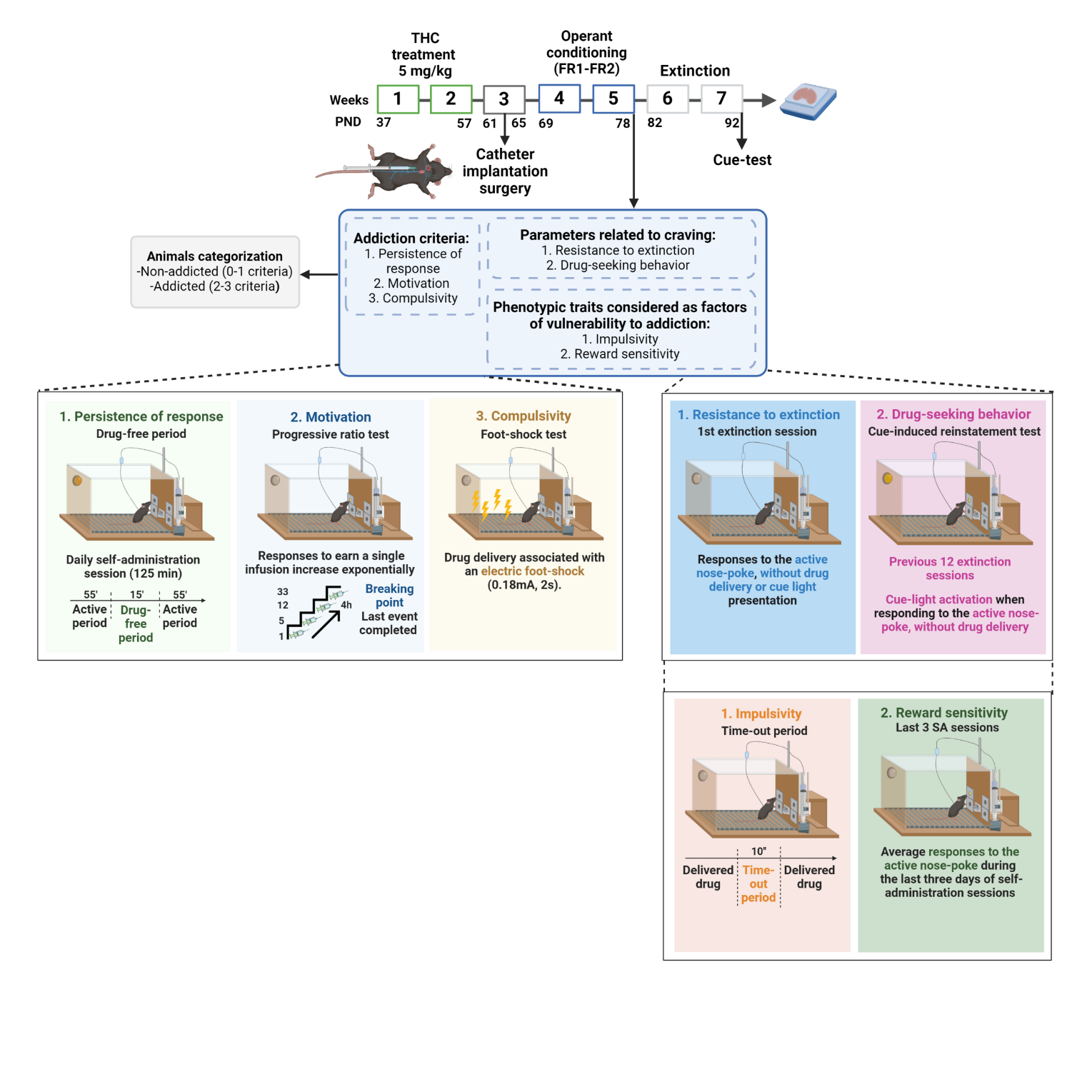


**SUPPLEMENTARY FIGURE 3.** Complete protocol of the WIN 55,212-2 self-administration mouse model after adolescent THC pre-exposure.

## Supplementary Tables

| **FIGURE 1. THC administration during adolescence led to the development of an addictive-like phenotype after WIN 55,212-2 operant self-administration in adult mice.** | | | | |
| --- | --- | --- | --- | --- |
| **Figure number** | **Statistical analysis** | **Factor name** | **Statistic value** | **P-value** |
| Fig. 1B | Repeated measures ANOVA | FR1 (Sessions 1-5)  Session  Treatment x Session  Treatment | *F*(4, 112)= 4.35  *F*(4, 112)= 0.11  *F*(1, 28)= 0.00 | p<0.01  n.s.  n.s. |
|  |  | FR2 (Sessions 6-10)  Session  Treatment x Session  Treatment | *F*(4,112)= 2.06  *F*(4,112)= 0.94  *F*(1, 28)= 1.00 | n.s.  n.s.  n.s. |
| Fig. 1  C-E | Shapiro-Wilk | Vehicle  Persistence  Motivation  Compulsivity  THC  Persistence  Motivation  Compulsivity | *S-W=* 0.90  *S-W=* 0.89  *S-W=* 0.69  *S-W=* 0.66  *S-W=* 0.89  *S-W=* 0.78 | n.s.  n.s.  p<0.001  p<0.001  n.s.  p<0.01 |
|  | Mann-Whitney U | Persistence  Motivation  Compulsivity | *U=*105,500  *t=* 0.87  *U=*110,000 | n.s.  n.s.  n.s. |
|  | t-test (equal variances assumed) |  |  |  |
|  | Mann-Whitney U |  |  |  |
| Fig. 1F | Chi square | Treatment | C-S= 0.019 | n.s. |
| Fig. 1  G-I | Shapiro-Wilk | NA Vehicle  Persistence  Motivation  Compulsivity  A Vehicle  Persistence  Motivation  Compulsivity  NA THC  Persistence  Motivation  Compulsivity  A THC  Persistence  Motivation  Compulsivity | *S-W=*0.96  *S-W=*0.91  *S-W=*0.65  *S-W=*0.98  *S-W=*0.85  *S-W=*0.88  *S-W=*0.83  *S-W=*0.84  *S-W=*0.68  *S-W=*0.75  *S-W=*0.86  *S-W=*0.55 | n.s.  n.s.  p<0.0001  n.s.  n.s.  n.s.  p<0.05  n.s.  p<0.001  p<0.05  n.s.  p<0.001 |
|  | t.test (equal variances assumed) | NA vs A Vehicle  Persistence  Motivation  Compulsivity  NA vs A THC  Persistence  Motivation  Compulsivity | *t=*-2.72  *t=*-1.33  *U=*14,000  *U=*12,500  *t=*-1.97  *U=*9,500 | p<0.05  n.s.  n.s.  n.s.  n.s.  n.s. |
|  | Mann-Whitney U |  |  |  |
|  | t.test (equal variances assumed) |  |  |  |
|  | Mann-Whitney U |  |  |  |
| Fig. 1  J-L | Pearson correlation | Vehicle  Persistence and addiction criteria  Motivation and addiction criteria  Compulsivity and addiction criteria  THC  Persistence and addiction criteria  Motivation and addiction criteria  Compulsivity and addiction criteria | *r=* 0.69  *r=* 0.51  *r=* 0.66  *r=* 0.46  *r=* 0.63  *r=* 0.31 | p<0.01  p<0.05  p<0.01  n.s.  p<0.05  n.s. |

| **FIGURE 2. THC administration during adolescence favored the parameters related with craving and the phenotypic vulnerability traits to addiction-like behavior after WIN 55,212-2 operant self-administration in adult mice.** | | | | |
| --- | --- | --- | --- | --- |
| **Figure number** | **Statistical analysis** | **Factor name** | **Statistic value** | **P-value** |
| Fig. 2A | Repeated measures ANOVA | Active levers  Session  Treatment x Session  Treatment VEH vsTHC  DMS: Actives vehicle vs. Actives THC  DMS: Actives vehicle vs. Inactives vehicle  DMS: Actives vehicle vs. Inactives THC  DMS: Inactives vehicle vs. Inactives THC  DMS: Actives THC vs. Inactives vehicle  DMS: Actives THC vs. Inactives THC | *F*(11, 528)= 5.68  *F*(11, 528)= 1.93  *F*(1, 48)= 15.26 | p<0.001  p<0.05  p<0.001  n.s.  p<0.001  p<0.001  n.s.  p<0.001  p<0.001 |
| Fig.  2B-C, 2H-I | Shapiro-Wilk | Vehicle  Resistance to extinction  Drug-seeking behaviour  Impulsivity  Reward sensitivity  THC  Resistance to extinction  Drug-seeking behavior  Impulsivity  Reward sensitivity | *S-W=*0.94  *S-W=*0.89  *S-W=*0.94  *S-W=*0.94  *S-W=*0.94  *S-W=*0.97  *S-W=*0.75  *S-W=*0.87 | n.s.  n.s.  n.s.  n.s.  n.s  n.s.  p<0.01  p<0.05 |
|  | t.test (equal variances assumed | Resistance to extinction  Drug-seeking behavior  Impulsivity  Impulsivity  Reward sensitivity | *t=*-2.18  *t=*0.89  *U=*70,000  *t=*--2.17  *U=*105,500 | p<0.05  n.s.  n.s.  p<0.05  n.s. |
|  | Mann-Whitney U |  |  |  |
|  | t.test (equal variances assumed) |  |  |  |
|  | Mann-Whitney U |  |  |  |
| Fig.  2D-E,  2J-K | Shapiro-Wilk | Vehicle NA  Resistance to extinction  Drug-seeking behaviour  Impulsivity  Reward sensitivity  Vehicle A  Resistance to extinction  Drug-seeking behaviour  Impulsivity  Reward sensitivity  THC NA  Resistance to extinction  Drug-seeking behaviour  Impulsivity  Reward sensitivity  THC A  Resistance to extinction  Drug-seeking behaviour  Impulsivity  Reward sensitivity | *S-W=*0.96  *S-W=*0.81  *S-W=*0.93  *S-W=*0.91  *S-W=*0.93  *S-W=*0.87  *S-W=*0.87  *S-W=*0.84  *S-W=*0.95  *S-W=*0.94  *S-W=*0.75  *S-W=*0.32  *S-W=*0.90  *S-W=*0.88  *S-W=*0.96  *S-W=*0.84 | n.s.  p<0.05  n.s.  n.s.  n.s.  n.s.  n.s.  p<0.001  n.s.  n.s.  p<0.01  p<0.01  n.s.  n.s.  n.s.  n.s. |
|  | Two–way ANOVA | Resistance to extinction  Phenotype (NA-A)  Treatment (VEH-THC)  Interaction (NA-A* VEH-THC) | *F*(1, 25)= 12.52  *F*(1, 25)= 6.872  *F*(1, 25)= 1.998 | p<0.01  p<0.05  n.s. |
|  | Mann-Whitney U | Vehicle NA vs A  Drug-seeking behaviour    Impulsivity  Reward sensitivity  THC NA vs A  Drug-seeking behaviour  Impulsivity  Reward sensitivity | *U=*10,000  *t=*-0.96  *U=*10,000  *t=*-0.26  *U=6*,500  *U=8*,000 | p<0.05  n.s.  p<0.05  n.s.  p<0.05  n.s. |
|  | t.test (equal variances assumed |  |  |  |
|  | Mann-Whitney U |  |  |  |
|  | t.test (equal variances assumed |  |  |  |
|  | Mann-Whitney U |  |  |  |
| Fig.  2F-G,  2L-M | Pearson correlation | Vehicle  Resistance to ext. and addiction criteria  Drug-seeking and addiction criteria  Impulsivity and addiction criteria  Reward sensitivity and addiction criteria  THC  Resistance to ext. and addiction criteria  Drug-seeking and addiction criteria Impulsivity and addiction criteria  Reward sensitivity and addiction criteria | *r=* 0.62  *r=* 0.31  *r=* 0.31  *r=* 0.59  *r=* 0.20  *r=* 0.26  *r=* 0.57  *r=* 0.41 | p<0.01  n.s.  n.s.  p<0.05  n.s.  n.s.  p<0.05  n.s. |

| **FIGURE 3. Characterization of Low-Impulsive (LI) and High-Impulsive (HI) subgroups.** | | | | |
| --- | --- | --- | --- | --- |
| **Figure number** | **Statistical analysis** | **Factor name** | **Statistic value** | **P-value** |
| Fig. 3A | Chi square | Treatment | C-S= 2.571 | n.s. |
| Fig. 3B | Repeated measures ANOVA | FR1 (Sessions 1-5)  Session  Treatment x Session  Treatment VEH vs THC  DMS: HI vehicle vs. LI vehicle  DMS: HI vehicle vs. LI THC  DMS: HI vehicle vs. HI THC  DMS: HI THC vs. LI vehicle  DMS: HI THC vs. LI THC  DMS: LI vehicle vs. LI THC | *F*(4, 104)= 3.35  *F*(4, 104)= 1.92  *F*(1, 26)= 3.46 | p<0.05  n.s.  p<0.05  n.s.  n.s.  p<0.05  p<0.05  p<0.01  n.s. |
|  |  | FR2 (Sessions 6-10)  Session  Treatment x Session  Treatment VEH vs THC  DMS: HI vehicle vs. LI vehicle  DMS: HI vehicle vs. LI THC  DMS: HI vehicle vs. HI THC  DMS: HI THC vs. LI vehicle  DMS: HI THC vs. LI THC  DMS: LI vehicle vs. LI THC | *F*(5, 130)= 1.79  *F*(5, 130)= 0.80  *F*(1, 26)= 7.86 | n.s.  n.s.  p<0.001  n.s.  n.s.  p<0.01  p<0.001  p<0.001  n.s. |
| Fig. 3C | Repeated measures ANOVA | FR1 (Sessions 1-5)  Session  Treatment x Session  Treatment VEH vs THC  DMS: HI vehicle vs. LI vehicle  DMS: HI vehicle vs. LI THC  DMS: HI vehicle vs. HI THC  DMS: HI THC vs. LI vehicle  DMS: HI THC vs. LI THC  DMS: LI vehicle vs. LI THC | *F*(4, 104)= 6.18  *F*(4, 104)= 3.34  *F*(1, 26)= 3.44 | p<0.001  p<0.01  p<0.05  n.s.  n.s.  n.s.  n.s.  p<0.01  n.s. |
|  |  | FR2 (Sessions 6-10)  Session  Treatment x Session  Treatment VEH vs THC  DMS: HI vehicle vs. LI vehicle  DMS: HI vehicle vs. LI THC  DMS: HI vehicle vs. HI THC  DMS: HI THC vs. LI vehicle  DMS: HI THC vs. LI THC  DMS: LI vehicle vs. LI THC | *F*(4, 104)= 2.64  *F*(4, 104)= 1.72  *F*(1, 26)= 12.73 | n.s.  n.s.  p<0.001  n.s.  p<0.05  p<0.01  p<0.001  p<0.001  n.s. |
| Fig.  3D-F | Shapiro-Wilk | Vehicle LI  Persistence of response  Motivation  Compulsivity  Vehicle HI  Persistence of response  Motivation  Compulsivity  THC LI  Persistence of response  Motivation  Compulsivity  THC HI  Persistence of response  Motivation  Compulsivity | *S-W=*0.94  *S-W=*0.85  *S-W=*0.66  *S-W=*0.86  *S-W=*0.76  *S-W=*0.81  *S-W=*0.66  *S-W=*0.77  *S-W=*0.74  *S-W=*0.91  *S-W=*0.55  *S-W=*0.88 | n.s.  n.s.  p<0.001  n.s  p<0.05  p<0.05  p<0.001  p<0.05  p<0.01  n.s  p<0.001  n.s. |
|  | t.test (equal variances assumed | Vehicle LI vs Vehicle HI  Persistence of response  Motivation  Compulsivity  THC LI vs THC HI  Persistence of response  Motivation  Compulsivity  Vehicle LI vs THC LI  Persistence of response  Motivation  Compulsivity  Vehicle HI vs THC HI  Persistence of response  Motivation  Compulsivity | *t=*-0.47  *U=*18.000  *U=*21,500  *U=18*,000  *U=*7,000  *U=*14,000  *U=28*,000  *U=*21,500  *U=*32,500  *t=*0.46  *U=*21,000  *U=*20,000 | n.s.  n.s.  n.s.  n.s.  p<0.05  n.s.  n.s.  n.s.  n.s.  n.s.  n.s.  n.s. |
|  | Mann-Whitney U |  |  |  |
|  | t.test (equal variances assumed |  |  |  |
|  | Mann-Whitney U |  |  |  |
| Fig.  3G-J | Shapiro-Wilk | Vehicle LI  Resistance to extinction  Drug-seeking behaviour  Impulsivity  Reward sensitivity  Vehicle HI  Resistance to extinction  Drug-seeking behaviour  Impulsivity  Reward sensitivity  THC LI  Resistance to extinction  Drug-seeking behaviour  Impulsivity  Reward sensitivity  THC HI  Resistance to extinction  Drug-seeking behaviour  Impulsivity  Reward sensitivity | *S-W=*0.86  *S-W=*0.85  *S-W=*0.92  *S-W=*0.93  *S-W=*0.87  *S-W=*0.77  *S-W=*0.77  *S-W=*0.90  *S-W=*0.93  *S-W=*0.95  *S-W=*0.80  *S-W=*0.91  *S-W=*0.96  *S-W=*0.95  *S-W=*0.86  *S-W=*0.81 | n.s.  n.s.  n.s.  n.s.  n.s.  p<0.05  p<0.05  n.s.  n.s.  n.s.  p<0.05  n.s.  n.s.  n.s.  n.s.  n.s. |
|  | t.test (equal variances assumed | Vehicle LI vs Vehicle HI  Resistance to extinction  Drug-seeking behaviour  Impulsivity  THC LI vs THC HI  Resistance to extinction  Drug-seeking behaviour  Impulsivity  Vehicle LI vs THC LI  Resistance to extinction  Drug-seeking behaviour  Impulsivity  Vehicle HI vs THC HI  Resistance to extinction  Drug-seeking behaviour  Impulsivity | *t=-1.31*  *U=22,000*  *U=*,000  *t=-1.31*  *t=-0.85*  *U=*,000  *t=*1.25  *t=*0.60  *U=15,000*  *t=*1.90  *U=13,000*  *U=,000* | n.s.  n.s.  p<0.001  n.s.  n.s.  p<0.001  n.s.  n.s.  p<0.05  n.s.  n.s.  p<0.001 |
|  | Mann-Whitney U |  |  |  |
|  | t.test (equal variances assumed |  |  |  |
|  | Mann-Whitney U |  |  |  |
|  | t.test (equal variances assumed |  |  |  |
|  | Mann-Whitney U |  |  |  |
|  | t.test (equal variances assumed |  |  |  |
|  | Mann-Whitney U |  |  |  |
|  | Two-way ANOVA | Reward sensitivity  Phenotype (LI-HI)  Treatment (VEH-THC)  Interaction (LI-HI* VEH-THC)  Tukey’s multiple comparisons test:  LI VEH vs. HI VEH  LI THC vs. HI THC  LI VEH vs. LI THC  HI VEH vs. HI THC  LI VEH vs. HI THC  LI THC vs. HI VEH | *F*(1, 26)= 39.38  *F*(1, 26)= 5.194  *F*(1, 26)= 7.314 | p<0.001  p<0.05  p<0.05  n.s.  p<0.001  n.s.  p<0.05  p<0.001  p<0.05 |

| **FIGURE 6. *drd1*, *drd2*, *adora2a* and *cnr1* gene expression in the medial prefrontal cortex (mPFC) and nucleus accumbens (NAc) after adolescent THC exposure in WIN 55,212-2 operant self-administering adult mice.** | | | | |
| --- | --- | --- | --- | --- |
| **Figure number** | **Statistical analysis** | **Factor name** | **Statistic value** | **P-value** |
| Fig. 6  A-D | Shapiro-Wilk | Vehicle  CB1R  D1R  D2R  Adora2AR  THC  CB1R  D1R  D2R  Adora2AR | *S-W=* 0.89  *S-W=* 0.93  *S-W=* 0.76  *S-W=* 0.84  *S-W=* 0.96  *S-W=* 0.91  *S-W=* 0.80  *S-W=* 0.88 | n.s.  n.s.  p<0.01  p<0.05  n.s.  n.s.  p<0.05  n.s. |
|  | t-test (equal variances assumed) | Vehicle vs THC  CB1R  D1R  D2R  Adora2AR | *t=* -0.21  *t=* -0.65  *U=* 65.000  *U=* 72.500 | n.s.  n.s.  n.s.  n.s. |
|  | Mann-Whitney U |  |  |  |
| Fig. 6  E-H | Shapiro-Wilk | Vehicle NA  CB1R  D1R  D2R  Adora2AR  Vehicle A  CB1R  D1R  D2R  Adora2AR  THC NA  CB1R  D1R  D2R  Adora2AR  THC A  CB1R  D1R  D2R  Adora2AR | *S-W=* 0.82  *S-W=* 0.88  *S-W=* 0.82  *S-W=* 0.93  *S-W=* 0.89  *S-W=* 0.95  *S-W=* 0.92  *S-W=* 0.90  *S-W=* 0.90  *S-W=* 0.92  *S-W=* 0.78  *S-W=* 0.87  *S-W=* 0.94  *S-W=* 1.00  *S-W=* 0.91  *S-W=* 0.87 | p<0.05  n.s.  p<0.05  n.s.  n.s.  n.s.  n.s.  n.s.  n.s.  n.s.  p<0.05  n.s.  n.s.  n.s.  n.s.  n.s. |
|  | Mann-Whitney U | Vehicle NA vs A  CB1R  D1R  D2R  Adora2AR  THC NA vs A  CB1R  D1R  D2R  Adora2AR  Vehicle NA vs THC NA  CB1R  D1R  D2R  Adora2AR  Vehicle A vs THC A  CB1R  D1R  D2R  Adora2AR | *U=* 17,000  *t=* 0.15  *U=6,000*  *t=* 1.39  *t=* 1.95  *t=* -0.68  *U=*11,000  *t=* -1.22  *U=1*4,000  *t=* -0.14  *U=*25,000  *t=* 0.84  *t=* 1.38  *t=* 0.75  *t=*-1.97  *t=* -1.71 | n.s.  n.s.  p<0.05  n.s.  n.s.  n.s.  n.s.  n.s.  n.s.  n.s.  n.s.  n.s.  n.s.  n.s.  n.s.  n.s |
|  | t-test (equal variances assumed) |  |  |  |
|  | Mann-Whitney U |  |  |  |
|  | t-test (equal variances assumed) |  |  |  |
|  | Mann-Whitney U |  |  |  |
|  | t-test (equal variances assumed) |  |  |  |
|  | Mann-Whitney U |  |  |  |
|  | t-test (equal variances assumed) |  |  |  |
|  | Mann-Whitney U |  |  |  |
|  | t-test (equal variances assumed) |  |  |  |
| Fig. 6  I-L | Shapiro-Wilk | Vehicle  CB1R  D1R  D2R  Adora2AR  THC  CB1R  D1R  D2R  Adora2AR | *S-W=* 0.92  *S-W=* 0.91  *S-W=* 0.90  *S-W=* 0.89  *S-W=* 0.93  *S-W=* 0.96  *S-W=* 0.94  *S-W=* 0.96 | n.s.  n.s.  n.s.  n.s.  n.s.  n.s.  n.s.  n.s. |
|  | t-test (equal variances assumed) | Vehicle vs THC  CB1R  D1R  D2R  Adora2AR | *t=* -0.33  *t=* 0.05  *t=* 2.60  *t=* 2.83 | n.s.  n.s.  p<0.05  p<0.01 |
| Fig. 6  M-P | Shapiro-Wilk | Vehicle NA  CB1R  D1R  D2R  Adora2AR  Vehicle A  CB1R  D1R  D2R  Adora2AR  THC NA  CB1R  D1R  D2R  Adora2AR  THC A  CB1R  D1R  D2R  Adora2AR | *S-W=* 0.96  *S-W=* 0.91  *S-W=* 0.87  *S-W=* 0.90  *S-W=* 0.89  *S-W=* 0.87  *S-W=* 0.88  *S-W=* 0.80  *S-W=* 0.94  *S-W=* 0.95  *S-W=* 0.90  *S-W=* 0.99  *S-W=* 0.76  *S-W=* 0.99  *S-W=* 0.93  *S-W=* 0.95 | n.s.  n.s.  n.s.  n.s.  n.s.  n.s.  n.s.  n.s.  n.s.  n.s.  n.s.  n.s.  p<0.05  n.s.  n.s.  n.s. |
|  | t-test (equal variances assumed) | Vehicle NA vs A  CB1R  D1R  D2R  Adora2AR  THC NA vs A  CB1R  D1R  D2R  Adora2AR  Vehicle NA vs THC NA  CB1R  D1R  D2R  Adora2AR  Vehicle A vs THC A  CB1R  D1R  D2R  Adora2AR | *t=* 1.30  *t=* 0.52  *t=* 0.17  *t=* 0.28  *U=* 4,000  *t=* 1.59  *t=* 0.60  *t=* 0.81  *t=* 0.09  *t=* -0.61  *t=* 1.63  *t=* 1.71  *U=* 10.000  *t=* 1.04  *t=* 2.44  *t=* 3.10 | n.s.  n.s.  n.s.  n.s.  n.s.  n.s.  n.s.  n.s.  n.s.  n.s.  n.s.  n.s.  n.s.  n.s.  p<0.05  p<0.05 |
|  | Mann-Whitney U |  |  |  |
|  | t-test (equal variances assumed) |  |  |  |
|  | Mann-Whitney U |  |  |  |
|  | t-test (equal variances assumed) |  |  |  |

| **FIGURE 7. *drd1*, *drd2*, *adora2a* and *cnr1* gene expression in the dorsal striatum (DS) and hippocampus (HPC) after adolescent THC exposure in WIN 55,212-2 operant self-administering adult mice.** | | | | |
| --- | --- | --- | --- | --- |
| **Figure number** | **Statistical analysis** | **Factor name** | **Statistic value** | **P-value** |
| Fig. 7  A-D | Shapiro-Wilk | Vehicle  CB1R  D1R  D2R  Adora2AR  THC  CB1R  D1R  D2R  Adora2AR | *S-W=* 0.96  *S-W=* 0.91  *S-W=* 0.98  *S-W=* 0.95  *S-W=* 0.99  *S-W=* 0.81  *S-W=* 0.94  *S-W=* 0.98 | n.s.  n.s.  n.s.  n.s.  n.s.  p<0.05  n.s.  n.s. |
|  | t-test (equal variances assumed) | Vehicle vs THC  CB1R  D1R  D2R  Adora2AR | *t=* 0.78  *U=* 77.000  *t=* 0.64  *t=* 0.84 | n.s.  n.s.  n.s.  n.s. |
|  | Mann-Whitney U |  |  |  |
|  | t-test (equal variances assumed) |  |  |  |
| Fig. 7  E-H | Shapiro-Wilk | Vehicle NA  CB1R  D1R  D2R  Adora2AR  Vehicle A  CB1R  D1R  D2R  Adora2AR  THC NA  CB1R  D1R  D2R  Adora2AR  THC A  CB1R  D1R  D2R  Adora2AR | *S-W=* 0.94  *S-W=* 0.91  *S-W=* 0.97  *S-W=* 0.90  *S-W=* 0.87  *S-W=* 0.85  *S-W=* 0.92  *S-W=* 0.94  *S-W=* 0.99  *S-W=* 0.90  *S-W=* 0.86  *S-W=* 1.00  *S-W=* 0.97  *S-W=* 0.78  *S-W=* 0.99  *S-W=* 1.00 | n.s.  n.s.  n.s.  n.s.  n.s.  n.s.  n.s.  n.s.  n.s.  n.s.  n.s.  n.s.  n.s.  n.s.  n.s.  n.s. |
|  | t-test (equal variances assumed) | Vehicle NA vs A  CB1R  D1R  D2R  Adora2AR  THC NA vs A  CB1R  D1R  D2R  Adora2AR  Vehicle NA vs THC NA  CB1R  D1R  D2R  Adora2AR  Vehicle A vs THC A  CB1R  D1R  D2R  Adora2AR | *t=* 1.42  *t=* 0.59  *t=* 1.27  *t=* 0.12  *t=* 0.42  *t=* 0.50  *t=* 0.83  *t=* 0.58  *t=* 0.15  *t=* 0.25  *t=* 0.54  *t=* -0.08  *t=* -0.12  *t=* 0.34  *t=* 0.75  *t=* 0.41 | n.s.  n.s.  n.s.  n.s.  n.s.  n.s.  n.s.  n.s.  n.s.  n.s.  n.s.  n.s.  n.s.  n.s.  n.s.  n.s. |
| Fig. 7  I-L | Shapiro-Wilk | Vehicle  CB1R  D1R  D2R  Adora2AR  THC  CB1R  D1R  D2R  Adora2AR | *S-W=* 0.93  *S-W=* 0.93  *S-W=* 0.87  *S-W=* 0.80  *S-W=* 0.90  *S-W=* 0.92  *S-W=* 0.91  *S-W=* 0.78 | n.s.  n.s.  n.s.  p<0.01  n.s.  n.s.  n.s.  p<0.05 |
|  | t-test (equal variances assumed) | Vehicle vs THC  CB1R  D1R  D2R  Adora2AR | *t=* 0.005  *t=* 0.80  *t=* 2.54  *U=* 31.000 | n.s.  n.s.  p<0.01  p<0.05 |
|  | Mann-Whitney U |  |  |  |
| Fig. 7  M-P | Shapiro-Wilk | Vehicle NA  CB1R  D1R  D2R  Adora2AR  Vehicle A  CB1R  D1R  D2R  Adora2AR  THC NA  CB1R  D1R  D2R  Adora2AR  THC A  CB1R  D1R  D2R  Adora2AR | *S-W=* 0.92  *S-W=* 0.95  *S-W=* 0.70  *S-W=* 0.65  *S-W=* 0.91  *S-W=* 0.88  *S-W =* 0.84  *S-W=* 0.94  *S-W=* 0.68  *S-W=* 0.92  *S-W=* 0.97  *S-W=* 0.73  *S-W=* 0.96  *S-W=* 0.89  *S-W=* 1.00  *S-W=* 0.96 | n.s.  n.s.  p<0.01  p<0.001  n.s.  n.s.  n.s.  n.s.  p<0.01  n.s.  n.s.  p<0.05  n.s.  n.s.  n.s.  n.s. |
|  | t-test (equal variances assumed) | Vehicle NA vs A  CB1R  D1R  D2R  Adora2AR  THC NA vs A  CB1R  D1R  D2R  Adora2AR  Vehicle NA vs THC NA  CB1R  D1R  D2R  Adora2AR  Vehicle A vs THC A  CB1R  D1R  D2R  Adora2AR | *t=* 1.86  *t=* -2.56  *U=* 6.000  *U=* 8.000  *U=* 8.000  *t=* -1.63  *t=* 0.76  *U=* 5,000  *U= 26*.000  *t=* 0.14  *U=* 14.000  *U=* 24.000  *U=* 12.000  *t=* 0.86  *t=* 3.18  *t=* 3.06 | n.s.  n.s.  n.s.  n.s.  n.s.  n.s.  n.s.  n.s.  n.s.  n.s.  n.s.  n.s.  n.s.  n.s.  p<0.05  p<0.05 |
|  | Mann-Whitney U |  |  |  |
|  | t-test (equal variances assumed) |  |  |  |
|  | Mann-Whitney U |  |  |  |
|  | t-test (equal variances assumed) |  |  |  |
|  | Mann-Whitney U |  |  |  |
|  | t-test (equal variances assumed) |  |  |  |

| **SUPPLEMENTARY FIGURE 1. THC pre-treatment did not produce major long-term behavioral or somatic alterations.** | | | | |
| --- | --- | --- | --- | --- |
| **Figure number** | **Statistical analysis** | **Factor name** | **Statistic value** | **P-value** |
| Fig. Supp. 1A | Repeated measures ANOVA | BW  Session  Treatment x Session  Treatment | *F*(15, 390)= 58.33  *F*(15, 390)= 0.88  *F*(1, 26)= 1.46 | p<0.001  n.s.  n.s. |
| Fig. Supp. 1B | Repeated measures ANOVA | BW  Session  Treatment x Session  Treatment | *F*(10, 260)= 137.5  *F*(10, 260)= 2.86  *F*(1, 26)= 1.63 | p<0.001  p<0.05  n.s. |
| Fig. Supp. 1C | Repeated measures ANOVA | FI  Session  Treatment x Session  Treatment | *F*(7, 182)= 6.07  *F*(7, 182)= 0.79  *F*(1, 26)= 0.02 | p<0.01  n.s.  n.s. |
| Fig. Supp. 1D | Repeated measures ANOVA | Kinetics of total activity  Time  Treatment x Time  Treatment | *F*(11, 308)= 37.96  *F*(11, 308)= 0.80  *F*(1, 28)= 1.50 | p<0.001  n.s.  n.s. |

| **SUPPLEMENTARY FIGURE 2. Extinction pattern of the first extinction session.** | | | | |
| --- | --- | --- | --- | --- |
| **Figure number** | **Statistical analysis** | **Factor name** | **Statistic value** | **P-value** |
| Fig. Supp. 2 | Shapiro-Wilk | Vehicle NA  10min  20min  30min  40min  50min  60min  70min  80min  90min  100min  110min  120min  Vehicle A  10min  20min  30min  40min  50min  60min  70min  80min  90min  100min  110min  120min  THC NA  10min  20min  30min  40min  50min  60min  70min  80min  90min  100min  110min  120min  THC A  10min  20min  30min  40min  50min  60min  70min  80min  90min  100min  110min  120min | *S-W=* 0.96  *S-W=* 0.91  *S-W=* 0.90  *S-W=* 0.86  *S-W=* 0.75  *S-W=* 0.94  *S-W=* 0.88  *S-W=* 0.77  *S-W=* 0.81  *S-W=* 0.76  *S-W=* 0.82  *S-W=* 0.80  *S-W=* 0.90  *S-W=* 0.88  *S-W=* 0.90  *S-W=* 0.91  *S-W=* 0.79  *S-W=* 0.83  *S-W=* 0.85  *S-W=* 0.91  *S-W=* 0.74  *S-W=* 0.84  *S-W=* 0.77  *S-W=* 0.90  *S-W=* 0.91  *S-W=* 0.84  *S-W=* 0.62  *S-W=* 0.87  *S-W=* 0.86  *S-W=* 0.87  *S-W=* 0.76  *S-W=* 0.72  *S-W=* 0.68  *S-W=* 0.83  *S-W=* 0.75  *S-W=* 0.77  *S-W=* 0.93  *S-W=* 0.98  *S-W=* 0.97  *S-W=* 0.87  *S-W=* 0.78  *S-W=* 0.87  *S-W=* 0.86  *S-W=* 0.96  *S-W=* 0.86  *S-W=* 0.95  *S-W=* 0.87  *S-W=* 0.82 | n.s.  n.s  n.s.  n.s.  p<0.01  n.s.  p<0.001  p<0.001  p<0.05  p<0.01  p<0.05  p<0.04  n.s.  n.s.  n.s.  n.s.  p<0.05  n.s.  n.s.  n.s.  p<0.05  n.s.  p<0.05  n.s.  n.s.  n.s.  p<0.001  n.s.  n.s.  n.s.  p<0.01  p<0.01  p<0.001  p<0.05  p<0.01  p<0.01  n.s.  n.s.  n.s.  n.s.  n.s.  n.s.  n.s.  n.s.  n.s.  n.s.  n.s.  n.s. |
|  | Friedman test | Vehicle NA  Vehicle A  THC NA  THC A | C-S=29.75  C-S=35.25  C-S=21.05  C-S=22.13 | p<0.01  p<0.001  p<0.05  p<0.05 |
|  | Mann-Whitney U | Vehicle NA vs Vehicle A  10min  20min  30min  40min  50min  60min  70min  80min  90min  100min  110min  120min  THC NA vs THC A  10min  20min  30min  40min  50min  60min  70min  80min  90min  100min  110mim  120min | *U*=11,000  *U*=7,500  *U*=11,500  *U*=18,000  *U*=17,000  *U*=20,500  *U*=20,500  *U*=23,000  *U*=4,500  *U*=16,500  *U*=19,500  *U*=15,000  *U*=17,500  *U*=17,000  *U*=16,500  *U*=12,500  *U*=18,500  *U*=21,000  *U*=12,500  *U*=14,500  *U*=19,000  *U*=20,000  *U*=17,000  *U*=21,000 | p<0.05  p<0.05  p<0.05  n.s.  n.s.  n.s.  n.s.  n.s.  p<0.01  n.s.  n.s.  n.s.  n.s.  n.s.  n.s.  n.s.  n.s.  n.s.  n.s.  n.s.  n.s.  n.s.  n.s.  n.s. |
